# The operculate micro land snails from the Alycaeinae (Caenogastropoda, Cyclophoridae) in Laos, with description of new species

**DOI:** 10.3897/zookeys.1290.198096

**Published:** 2026-08-25

**Authors:** Parin Jirapatrasilp, Khamla Inkhavilay, Chanvilay Somvongsa, Banchai Malavong, Teerangkul Janjai, Ekgachai Jeratthitikul, Natdanai Likhitrakarn, Ruttapon Srisonchai, Chirasak Sutcharit

**Affiliations:** 1 Animal Systematics Research Unit, Department of Biology, Faculty of Science, Chulalongkorn University, Bangkok 10330, Thailand Animal Systematics and Molecular Ecology Laboratory, Department of Biology, Faculty of Science, Mahidol University Bangkok Thailand; 2 Research and Academic Service Office, National University of Laos, P.O. Box 7322, Dongdok, Vientiane, Laos Animal Systematics Research Unit, Department of Biology, Faculty of Science, Chulalongkorn University Bangkok Thailand; 3 Animal Systematics and Molecular Ecology Laboratory, Department of Biology, Faculty of Science, Mahidol University, Bangkok 10400, Thailand Research and Academic Service Office, National University of Laos Vientiane Laos; 4 Program of Agriculture, Faculty of Agricultural Production, Maejo University, Chiang Mai 50290, Thailand Program of Agriculture, Faculty of Agricultural Production, Maejo University Chiang Mai Thailand; 5 Department of Biology, Faculty of Science, Khon Kaen University, Khon Kaen 40002, Thailand Department of Biology, Faculty of Science, Khon Kaen University Khon Kaen Thailand

**Keywords:** Cyclophoroidea, Gastropoda, Mollusca, taxonomy

## Abstract

This study reports on the operculate micro land snail species from the subfamily Alycaeinae collected during recent biodiversity surveys in the limestone-rich areas in Vientiane and Houaphanh provinces, Laos. Altogether eight species are reported, four of which are new to science and described herein based on their distinct shell morphology: *Dioryx feuangensis* Jirapatrasilp & Inkhavilay, **sp. nov**., *Dicharax feuangensis* Jirapatrasilp & Inkhavilay, **sp. nov**., *Dicharax pallgergelyi* Jirapatrasilp & Inkhavilay, **sp. nov**., and *Pincerna panhai* Jirapatrasilp & Inkhavilay, **sp. nov**. One nominal species, *Dioryx dongiensis*, is recorded from Laos for the first time. These discoveries highlight the importance of limestone karst ecosystems that contribute to Lao biodiversity, especially land malacofauna.

## Introduction

Lao People’s Democratic Republic or ‘Laos’, a landlocked country in mainland Southeast Asia, has received wider attention for biodiversity studies, leading to several discoveries of numerous new invertebrate species in the past few years (e.g., Chanabun et al. 2023; Hong et al. 2024; Likhitrakarn et al. 2024; Sriranan et al. 2025). Among these invertebrates are land snails, where more than 240 species have been recently compiled (Inkhavilay et al. 2019; Vanhdibay et al. 2023). Of this total, more than 120 new land snail species from several families have been discovered and described in the past decades, e.g., Diplommatinidae (Páll-Gergely and Grego 2020), Pupinidae (Páll-Gergely et al. 2015a; Páll-Gergely and Grego 2019), Camaenidae (Inkhavilay et al. 2017; Páll-Gergely et al. 2023a), Hypselostomatidae (Páll-Gergely et al. 2022, 2023b; Inkhavilay and Sutcharit 2024; Gojšina et al. 2025; Do et al. 2026; Janjai et al. 2026), Diapheridae (Jochum et al. 2020; Páll-Gergely et al. 2020c; Inkhavilay et al. 2024), Streptaxidae (Inkhavilay et al. 2016a; Do 2021; Vanhdibay et al. 2023; Sutcharit et al. 2025), Trochomorphidae (Inkhavilay et al. 2023), and Plectopylidae (Páll-Gergely et al. 2016a) (Table 1).

**Table 1.** Updated list of land snail taxa newly described or recorded from Laos since Inkhavilay et al. (2019). The modern locality names are given in square brackets.

**No**.	**Species**	**Type locality (record in Laos if the type locality is not in Laos)**	**References**
Family Cyclophoridae			
1	*Alycaeus goliath* Páll-Gergely, 2023	~ 4.5 km WNW of Mahaxay, ~ 37 km ENE of Thakhek (Muang Khammouane), Khammouane Province, Laos	Páll-Gergely (2023b)
2	*Cyclophorus thakhekensis* Thach & Huber, 2018	Thakhek, Khammouane Province, Laos	Thach (2018b)
3	*Cyclotus huberi* Thach, 2018	Kasi, Vientiane Province, Laos	Thach (2018b)
4	*Rhiostoma abletti* Thach, 2016	Northwest of Lai Chau city, on the way going to Paso, Lai Chau Province, North Vietnam (Houaphanh Province, Laos)	Thach (2016), Tongkerd et al. (2023)
5	*Rhiostoma anceyi* Tongkerd & Inkhavilay, 2023	Ban Pha Hom (village), Phoxay District, Vientiane Province, Laos	Tongkerd et al. (2023)
6	*Rhiostoma franzhuberi* Thach, 2023	Vieng Xai, Houaphanh Province, Laos	Thach (2023)
7	*Rhiostoma houaphanhense* Thach & Huber, 2023	Vieng Xai, Houaphanh Province, Laos	Thach (2023)
8	*Rhiostoma laosense* Tongkerd & Inkhavilay, 2023	Wat Pa Pha (temple), Khamkeut District, Bolikhamsai Province, Laos	Tongkerd et al. (2023)
9	*Rhiostoma thachi* Huber, 2018	Thakhek, Khammouane Province, Laos	Thach (2018b), Tongkerd et al. (2023)
10	*Scabrina franzhuberi* Thach, 2020	Vang Vieng, Vientiane Province, Laos	Thach (2020)
11	*Scabrina huberorum* Thach, 2023	Phonsavan District, Xieng Khouang Province, Laos	Thach (2023)
Family Diplommatinidae			
12	*Arinia boucheti* Páll-Gergely, 2018	Phuom Laong, northern Laos	Páll-Gergely and Hunyadi (2018)
13	*Kontschania tetragyra* Páll-Gergely & Grego, 2020	Tham Nam Don Cave, Khammouane Province, Laos	Páll-Gergely and Grego (2020)
14	*Laotia christahemmenae christahemmenae* Páll-Gergely, 2014	Ba Vi National Park, Ha Noi Province, Vietnam (Luang Prabang Province, Laos)	Páll-Gergely (2014), Páll-Gergely and Hunyadi (2021)
Family Pollicariidae			
15	*Pollicaria huberi* Thach, 2018 [treated as junior synonym of *Pollicaria myersii* (Haines, 1855)]	Thakhek, Khammouane Province, Laos	Thach (2018b), Jirapatrasilp et al. (2022)
Family Pupinidae			
16	*Pseudopomatias franzhuberi* Thach, 2020	Luang Prabang, Luang Prabang Province, Laos	Thach (2020)
17	*Pupinella franzhuberi* Thach, 2020 [treated as junior synonym of *Pupinella mansuyi* (Dautzenberg & Fischer, 1908)]	Luang Prabang, Luang Prabang Province, Laos	Thach (2020), Jirapatrasilp et al. (2022)
18	*Rhaphaulus franzhuberi* Thach, 2021	Vang Vieng, Vientiane Province, Laos	Thach (2021)
19	*Vargapupa biheli viridis* Páll-Gergely & Grego, 2019	ca 4 km SW of Ban Khong, Xieng Khouang Province, Laos	Páll-Gergely and Grego (2019)
Family Achatinidae			
20	*Allopeas franzhuberi* Thach, 2021	Phonsavan District, Xieng Khouang Province, Laos	Thach (2021)
21	*Glessula huberi* Thach, 2018	Phonsavan District, Xieng Khouang Province, Laos	Thach (2018b)
22	*Prosopeas huberi* Thach, 2018	Phonsavan District, Xieng Khouang Province, Laos	Thach (2018b)
Family Diapheridae			
23	*Laoennea carychioides* Páll-Gergely, Reischütz & Maassen, 2020	Tham Pou Kham Cave, W of Vang Vieng, Vientiane Province, Laos	Páll-Gergely et al. (2020c), Vanhdibay et al. (2023)
24	*Laoennea renouardi* Jochum & Wackenheim, 2020	Tham Houey Yè (cave), 2.7 km W of Vang Vieng, Vientiane Province, Laos	Jochum et al. (2020), Vanhdibay et al. (2023)
25	*Sinoennea angustistoma* Páll-Gergely, Reischütz & Maassen, 2020	Limestone hill in the rice paddies E of road 1E about 300 m SE of Yommalath, NE of Thakhek, Khammouane Province, Laos	Páll-Gergely et al. (2020c), Vanhdibay et al. (2023)
26	*Sinoennea bounphanmyae* Inkhavilay & Vanhdibay, 2024	The limestone outcrop at Pha Thor Nor Kham Hills, Feuang District, Vientiane Province, Laos	Inkhavilay et al. (2024)
27	*Sinoennea houaphanica* Inkhavilay, Inthavong & Oulaiseng, 2024	Ban Lao, Xamtay District, Houaphanh Province, Laos	Inkhavilay et al. (2024)
28	*Sinoennea infantilis* Páll-Gergely & Grego, 2020	Earthquake Dome, Tham Don Cave, Khammouane Province, Laos	Páll-Gergely et al. (2020c), Vanhdibay et al. (2023)
29	*Sinoennea ljudmilena* Páll-Gergely, 2020	Tran Ninh, Pah Hia, Laos [Xieng Khouang Province, Laos]	Páll-Gergely et al. (2020c), Vanhdibay et al. (2023)
30	*Sinoennea otostoma* Páll-Gergely, Reischütz & Maassen, 2020	Tham Pou Kham (cave), west of Vang Vieng, Vientiane Province, Laos	Páll-Gergely et al. (2020c), Vanhdibay et al. (2023)
31	*Sinoennea variabilis* Páll-Gergely & Grego, 2020	ca 37 km east-northeast of Thakhek (Muang Khammouane), ca 4.5 km west-northwest of Mahaxai, Khammouane Province, Laos	Páll-Gergely et al. (2020c), Vanhdibay et al. (2023)
Family Streptaxidae			
32	*Discartemon feuangensis* Sutcharit & Inkhavilay, 2025	Pha Reung (limestone karst), Ban Pha Reung (village), Feuang District, Vientiane Province, Laos	Sutcharit et al. (2025)
33	*Haploptychius somsaki* Vanhdibay & Inkhavilay, 2023	Tam Kong Lor (cave), Ban Kong Lor (village), Khounkham District, Khammouane Province, Laos	Vanhdibay et al. (2023)
34	*Indoartemon franzhuberi* Thach, 2021 [replacement name of *Indoartemon huberi* Thach, 2018]	Thakhek, Khammouane Province, Laos	Thach (2018b, 2021), Vanhdibay et al. (2023)
35	*Perrottetia dugasti laosiana* Thach & Huber, 2020	Luang Prabang, Luang Prabang Province, Laos	Thach (2020), Vanhdibay et al. (2023)
36	*Perrottetia huberorum* Thach, 2021	Vieng Xai, Houaphanh Province, Laos	Thach (2021), Vanhdibay et al. (2023)
37	*Perrottetia thachorum* Huber, 2021	Vieng Xai, Houaphanh Province, Laos	Thach (2021), Vanhdibay et al. (2023)
38	*Stemmatopsis dolium* Do, 2021	Pa Kha Village, Xieng Nam Phan Commune, Khun District, Khouang Province, Laos	Do (2021)
Family Cerastidae			
39	*Cerastus franzhuberi* Thach, 2020^a^	Phongsali Province, Laos	Thach (2020)
Family Enidae			
40	*Apoecus huberi* (Thach, 2018)	Mahaxai, Thakhek, Khammouane Province, Laos	Thach (2018b), Páll-Gergely et al. (2020a)
41	*Mirus huberi* Thach, 2018 [treated as junior synonym of *Apoecus macrostoma* (Bavay & Dautzenberg, 1912)]	Phonsavan District, Xieng Khouang Province, Laos	Thach (2018b), Páll-Gergely et al. (2020a)
Family Hypselostomatidae			
42	*Anauchen grandiportus* Gojšina, Grego & Páll-Gergely, 2025	NE foot of Mount Pha Soung, Khammouane Province, Laos	Gojšina et al. (2025)
43	*Anauchen kozari* Páll-Gergely, 2023	Phou Xuang mountain,~ 1.5 km NE of Ban Lak Sip,~ 5 km SE of Luang Prabang, Luang Prabang province, Laos	Páll-Gergely (2023a), Gojšina et al. (2025)
44	*Angustopila bathyodon* Páll-Gergely & Hunyadi, 2023	43.4 km from Yommalath towards Nongchan (bridge on road #12), southern edge of Ban Khamhé, Khammouane Province, Laos	Páll-Gergely et al. (2023b)
45	*Angustopila bidentata* Páll-Gergely & Jochum, 2023	ca 35 km ENE of Thakhek (Muang Khammouane),~ 7 km WNW of Mahaxai, Khammouane Province, Laos	Páll-Gergely et al. (2023b)
46	*Angustopila cavicola* Páll-Gergely & Dumrongrojwattana, 2023	Phu Tham Pha Tang, Loei Province, Thailand (Tham Nathong (cave), Ban Nathong, 6.5 km southeast from centre of Na Mor towards Oudomxay, Oudomxay Province, Laos)	Páll-Gergely et al. (2023b)
47	*Angustopila coprologos coprologos* Páll-Gergely, Jochum & Hunyadi, 2022	Phu Phako, 15 km southeast + 4.5 km northeast (on a side road) towards centre of Lak Sao, Bolikhamsai Province, Laos	Páll-Gergely et al. (2022, 2023b)
48	*Angustopila coprologos uninodus* Páll-Gergely & Grego, 2023	NE foot of Pha Hông limestone massif 7 km N of Lak Sao, Bolikhamsai Province, Laos	Páll-Gergely et al. (2023b)
49	*Angustopila elevata* (Thompson & Upatham, 1997)	Doi Chiang Dao (mountain), 7 km west of Chiang Dao, Chiang Mai Province, Thailand (northern Laos)	Thompson and Upatham (1997), Páll-Gergely et al. (2023b)
50	*Angustopila fabella* Páll-Gergely & Hunyadi, 2015 [treated as senior synonym of *Angustopila singuladentis* Inkhavilay & Panha, 2016]	Cliffs north of Lenglei, north of the Nonggang Nature Reserve, Longzhou Xian, Chongzuo Shi, Guangxi, China (northern Laos)	Páll-Gergely et al. (2015b, 2023b), Inkhavilay et al. (2016b)
51	*Angustopila gracilis* Páll-Gergely & Hunyadi, 2023	Phu Phako, 15 km southeast from centre of Lak Sao, 4.5 km northeast on a side road, Bolikhamsai Province, Laos	Páll-Gergely et al. (2023b)
52	*Angustopila margaritarion* Páll-Gergely & Hunyadi, 2023	19.8 km southeast from centre of Vieng Phou Kha, south from Ban Phou Lek, Luang Namtha Province, Laos	Páll-Gergely et al. (2023b)
53	*Angustopila pusilla* Páll-Gergely & Hunyadi, 2023	Tham Khan Kham (cave), Phone Ngeun, 4.5 km west from centre of Vang Vieng, Vientiane Province, Laos	Páll-Gergely et al. (2023b)
54	*Angustopila steffeki* Páll-Gergely & Grego, 2023	Large spring lake at S foot of limestone massif 2 km NNE of Na Pavan village,~ 5 km from Phontan crossing at road 8, Bolikhamsai Province, Laos	Páll-Gergely et al. (2023b)
55	*Angustopila szekeresi* Páll-Gergely & Hunyadi, 2015	Cliffs at the southern edge of Jiaole Cun, Bama Xian, Hechi Shi, Guangxi, China (northern Laos)	Páll-Gergely et al. (2015b, 2023b)
56	*Bensonella cardiostoma* Gojšina, Vermeulen & Páll-Gergely, 2025	Nakay Village, Vang Vieng District, Vientiane Province, Laos	Gojšina et al. (2025)
57	*Bensonella expansa* Do, Nguyen & Dao, 2026	Limestone area in Nam Phan Commune, Khoune District, Xieng Khouang Province, Laos	Do et al. (2026)
58	*Bensonella hunlaman* Janjai, Inkhavilay, Jeratthitikul, Jirapatrasilp, Likhitrakarn, Srisonchai & Sutcharit, 2026	Limestone hills in Ban Na Dan, Feuang District, Vientiane Province, Laos	Janjai et al. (2026)
59	*Bensonella mitochondria* Gojšina, Vermeulen & Páll-Gergely, 2025	Base of forested limestone hill, Ban Nakok, Thakhek District, Khammouane Province, Laos	Gojšina et al. (2025)
60	*Bensonella perfecta* Gojšina & Páll-Gergely, 2025	ca 47 km ENE of Phou Khoun,~ 30 km WNW of Phonsavan, Xieng Khouang Province, Laos	Gojšina et al. (2025)
61	*Boysidia houaphanica* Inkhavilay & Sutcharit, 2024	Limestone outcrop at Ban Lao, Samtay District, Houaphanh Province, Laos	Inkhavilay and Sutcharit (2024)
62	*Clostophis infantilis* Páll-Gergely, 2020	ca 37 km ENE of Thakhek (Muang Khammouane),~ 4.5 km WNW of Mahaxai, Khammouane Province, Laos	Páll-Gergely et al. (2020b)
63	*Clostophis multiformis* Páll-Gergely & Reischütz, 2020	Limestone hill in rice fields east of the road 1E, 300 m SE Yommalath, NE Thakhek, Khammouane Province, Laos	Páll-Gergely et al. (2020b)
64	*Clostophis obtusus* Páll-Gergely & Grego, 2020	Earthquake Dome, Tham Nam Don Cave, Khammouane Province, Laos	Páll-Gergely et al. (2020b)
65	*Hypselostoma loei* Panha & Prateepasen, 2005	Limestone hills in Loei Province, Thailand (Limestone hills in Ban Pha Sung, Feuang District, Vientiane Province)	Panha and Prateepasen (2005), Janjai et al. (2026)
66	*Tonkinospira crassicostata* Páll-Gergely & Grego, 2019	500 m SE of village Ban Phondou, Khammouane Province, Laos	Páll-Gergely et al. (2019)
67	*Tonkinospira raxajacki* Páll-Gergely & Reischütz, 2019	Tham Xienliab (cave), NE Thakhek, Khammouane Province, Laos	Páll-Gergely et al. (2019)
68	*Tonkinospira suturata* Páll-Gergely & Grego, 2019	Earthquake Dome, Tham Nam Don Cave, Khammouane Province, Laos	Páll-Gergely et al. (2019)
Family Clausiliidae			
69	*Musaphaedusa hisanoi* Nordsieck, 2018	Nong Khiaw, in direction to Muang Ngoy, Luang Prabang Province, Laos	Nordsieck (2018)
70	*Oospira franzhuberi* Szekeres & Thach, 2018 [treated as junior synonym of *Oospira bolovenica* (Möllendorff, 1898)]	Attapeu Province, Laos	Thach (2018b), Páll-Gergely et al. (2020a)
Family Chronidae			
71	*Hemiglypta franzhuberi* Thach, 2018^b^	Mahaxai, 8 km from Vietnam borderline, Khammouane Province, Laos	Thach (2018b)
Family Dyakiidae			
72	*Quantula simonei* Thach & Huber, 2018	Laos	Thach (2018b)
73	*Quantula naggsi* Thach & Huber, 2020	Kasi, Vientiane Province, Laos	Thach (2020)
Family Trochomorphidae			
74	*Dendrotrochus huberi* Thach, 2020^c^	Luang Prabang, Luang Prabang Province, Laos	Thach (2020)
75	*Trochomorpha buotia* Inkhavilay, Sutcharit & Pholyotha, 2023	Tham Nang Rod (cave), Na-dan village, Yommalath District, Khammouane Province, Laos	Inkhavilay et al. (2023)
76	*Trochomorpha somsakpanhai* Inkhavilay, Sutcharit & Pholyotha, 2023	Tham Xang (cave), Thakhek District, Khammouane Province, Laos	Inkhavilay et al. (2023)
77	*Trochomorpha speirofascia* Inkhavilay, Sutcharit & Pholyotha, 2023	Par-Houak limestone, Vieng Swang village, Vieng Phoukha District, Luang Namtha Province, Laos	Inkhavilay et al. (2023)
78	*Trochomorpha thachi* Huber, 2020	Kasi District, Vientiane Province, Laos	Thach (2020), Inkhavilay et al. (2023)
Family Ariophantidae			
79	*Euplecta herosae* Thach & Huber, 2018 [treated as junior synonym of *Trichochloritis fouresi* (Morlet, 1886)]	Boloven Plateau, Laos	Thach (2018b), Páll-Gergely et al. (2020a)
80	*Euplecta hueae* Thach & Huber, 2018 [treated as junior synonym of *Ganesella rostrella* (Pfeiffer, 1863)]	Luang Prabang, Luang Prabang, Province, Laos	Thach (2018b), Páll-Gergely et al. (2020a)
81	*Euplecta huberi* Thach, 2018 [treated as junior synonym of *Ganesella emma* (Pfeiffer, 1863)]	Phonsavan District, Xieng Khouang Province, Laos	Thach (2018b), Páll-Gergely et al. (2020a)
82	*Hemiplecta franzhuberi* Thach, 2020 [treated as junior synonym of *Hemiplecta distincta* (Pfeiffer, 1850)]	Thakhek, Khammouane Province, Laos	Thach (2020), Sutcharit and Panha (2021)
Family Helicarionidae			
83	*Satiella bramvanderbijli* Thach & Huber, 2021	Vang Vieng, Vientiane Province, Laos	Thach (2021)
Family Camaenidae			
84	*Aegista stephanieclarkae* Thach & Huber, 2021	South Kasi, Vientiane Province, Laos	Thach (2021)
85	*Amphidromus anhduongae* Thach, 2020	Attapeu Province, Laos	Thach (2020)
86	*Amphidromus davidmonsecouri niarlingi* Thach, 2020 [treated as junior synonym of *Amphidromus smithii* Fulton, 1896]	Pakse, Champasak Province, Laos	Thach (2020), Do et al. (2024)
87	*Amphidromus donchani* Thach, 2019	Attapeu Province, Laos	Thach (2019b)
88	*Amphidromus eichhorsti* Thach, 2020 [treated as junior synonym of *Amphidromus cruentatus* (Morelet, 1875)]	North Laos	Thach (2020), Lee et al. (2022)
89	*Amphidromus gerberi bolovenensis* Thach & Huber, 2018 [treated as junior synonym of *Amphidromus cruentatus* (Morelet, 1875)]	Naoh, Boloven Plateau, Attapeu Province, Laos	Thach (2018b), Lee et al. (2022)
90	*Amphidromus goldbergi* Thach & Huber, 2018 [treated as junior synonym of *Amphidromus cruentatus* (Morelet, 1875)]	Salavan Province, Laos	Thach (2018b), Lee et al. (2022)
91	*Amphidromus hejingi arlinicoi* Thach, 2020	Pakse District, Champasak Province, Laos	Thach (2020)
92	*Amphidromus huynhanhi* Thach, 2019	Attapeu Province, Laos	Thach (2019a)
93	*Amphidromus koonpoi* Thach & Huber, 2018 [treated as junior synonym of *Amphidromus roseolabiatus* Fulton, 1896]	Thakhek, Khammouane Province, Laos	Thach (2018b), Páll-Gergely et al. (2020a)
94	*Amphidromus marki* Thach, 2021	Bolaven Plateau, Salavan Province, Laos	Thach (2021)
95	*Amphidromus nicoasiarum* Thach, 2021	Kaleum District, Sekong Province, Laos	Thach (2021)
96	*Amphidromus pengzhuoani* Thach, 2018 [treated as junior synonym of *Amphidromus cruentatus* (Morelet, 1875)]	Luang Namtha Province, Laos	Thach (2018a), Lee et al. (2022)
97	*Amphidromus poppei arnicoi* Thach, 2020	Pakse, Champasak Province, Laos	Thach (2020)
98	*Amphidromus severnsi luangprabangensis* Thach & Huber, 2020	10–12 km south of Luang Prabang, Luang Prabang Province, Laos	Thach (2020)
99	*Amphidromus thachorum* Huber, 2020	Attapeu Province, Laos	Thach (2020)
100	*Amphidromus truongkhoai* Thach, 2018 [treated as junior synonym of *Amphidromus flavus* (Pfeiffer, 1861)]	Savannakhet Province, Laos	Thach (2018b), Páll-Gergely et al. (2020a)
101	*Bellatrachia franzhuberi* (Thach, 2020)	Attapeu Province, Laos	Thach (2020), Páll-Gergely et al. (2026)
102	*Bouchetcamaena huberi* Thach, 2018 [treated as junior synonym of *Trichochloritis fouresi* (Morlet, 1886)]	South of Pakse, Champasak Province, Laos	Thach (2018b), Páll-Gergely et al. (2020a)
103	*Bouchetcamaena herosae* Thach & Huber, 2020	South of Kasi, Vientiane Province, Laos	Thach (2020)
104	*Bouchetcamaena maestrati* Thach & Huber, 2020	South of Kasi, Vientiane Province, Laos	Thach (2020)
105	*Bouchetcamaena thachi* Huber, 2018	South of Pakse, Champasak Province, Laos	Thach (2018b)
106	*Burmochloritis inkhavilayi* (Páll-Gergely, 2019) [replacement name of *Satsuma huberi* Thach, 2018]	Phonsavan District, Xieng Khouang Province, Laos	Thach (2018b), Páll-Gergely (2019), Páll-Gergely et al. (2023a)
107	*Burmochloritis laotica* Páll-Gergely & Neubert, 2023	Province of Tran Ninh, Pa Ka Tai, Laos [probably Ban Phoukatay, Xaisomboun Province]	Páll-Gergely et al. (2023a)
108	*Burmochloritis muratovi* Páll-Gergely, 2023	ca 7 km S of Luang Prabang, near Tad Thong waterfall, Luang Prabang Province, Laos	Páll-Gergely et al. (2023a)
109	*Burmochloritis nordsiecki* Páll-Gergely & Neubert, 2023	ca 6 km N of Phou Khoun, Luang Prabang Province, Laos	Páll-Gergely et al. (2023a)
110	*Burmochloritis paya* Páll-Gergely, 2023	Paya (Pa Hia?), Laos [probably refers to Ban Namthong, Longchaeng District, Xaisomboun Province]	Páll-Gergely et al. (2023a)
111	*Burmochloritis payella* Páll-Gergely, 2023	Paya (Pa Hia?), Laos	Páll-Gergely et al. (2023a)
112	*Calvigenia owengriffithsi* Thach & Huber, 2020^d^	Saou [= Ban Houei Saou], Luang Prabang Province, Laos	Thach (2020)
113	*Camaena franzhuberi laosiana* Thach, 2018	Khong Island, Champasak Province, Laos	Thach (2018b)
114	*Chloritis abletti* Thach & Huber, 2018	Mahaxai, Thakhek, Khammouane Province, Laos	Thach (2018b)
115	*Chloritis bolovenensis* Thach & Huber, 2021	Bolavan Plateau, South Laos	Thach (2021)
116	*Chloritis sliekeri* Thach & Huber, 2021	Vang Vieng area, Vientiane Province, Laos	Thach (2021)
117	*Chloritis thachi laosianus* Huber, 2018	Ban Nam Thone, Khammouane Province, Laos	Thach (2018b)
118	*Pseudobuliminus franzhuberi* Thach, 2018	Mahaxai, east of Thakhek, near Vietnam borderline, Khammouane Province, Laos	Thach (2018b)
119	*Pseudobuliminus thachi* Huber, 2018	Mahaxai, east of Thakhek, near Vietnam borderline, Khammouane Province, Laos	Thach (2018b)
120	*Trichochloritis huberi* (Thach, 2018)	Vang Vieng, Vientiane Province, Laos	Thach (2018b), Páll-Gergely et al. (2020a)
121	*Trichochloritis mussonena* Páll-Gergely, 2020 [replacement name of *Mussonena huberi* Thach, 2018]	Mahaxai, Thakhek, Khammouane Province, Laos	Thach (2018b), Páll-Gergely et al. (2020a)
122	*Vinatachea delsaerdti aurantia* (Thach & Huber, 2020)	6 km from borderline with Central Vietnam, Laos	Thach (2020), Páll-Gergely et al. (2026)

^a^The assignment of this species to *Cerastus* Martens, 1860 is doubtful, as the distribution of this genus is restricted to the Arabian Peninsula, East Africa, and West India (Schileyko 1998). ^b^The assignment of this species to *Hemiglypta* Möllendorff, 1893 is doubtful, as the distribution of this genus is mostly restricted to the Philippines and New Guinea (Schileyko 2003). ^c^The assignment of this species to *Dendrotrochus* Pilsbry, 1894 is doubtful, as the distribution of this genus is restricted to Papua New Guinea, Admiralty, Solomon, and Bismarck Islands (Schileyko 2002). ^d^The assignment of this species to *Calvigenia* Iredale, 1938 is doubtful, as the distribution of this genus is restricted to Queensland, Australia (Stanisic et al. 2010).

Of particular interest in the Laos malacofauna are the operculated land snails from the subfamily Alycaeinae of the Cyclophoridae. This land snail group is characterised by a sutural tube that connects to numerous perpendicular microtunnels, which open near the umbilicus (Páll-Gergely et al. 2016b, 2024). This subfamily contains more than 300 species from nine genera, and the members of this subfamily are highly diverse in mainland Southeast Asia (Páll-Gergely et al. 2020d, 2024; Jirapatrasilp et al. 2025; Thach 2025). Up to now, 14 species from the subfamily Alycaeinae have been reported from Laos (Table 2).

**Table 2.** List of species in the subfamily Alycaeinae reported from Laos. The type locality of each taxon is quoted verbatim from the original description, with modern locality names given in square brackets.

**Species**	**Type locality**	**Distribution records**	**References**
*Alycaeus goliath* Páll-Gergely, 2023	~ 37 km ENE of Thakhek (Muang Khammouane), ~ 4.5 km WNW of Mahaxay, Khammouane Province, Laos (17°25.956'N, 105°09.669'E)	The type locality only	Páll-Gergely (2023b)
*Alycaeus rolfbrandti* Maassen, 2006	Limestone hills 20 km E. of Takek, Laos	Khammouane and Bolikhamsai provinces, Laos	Maassen (2006), Inkhavilay et al. (2019), Páll-Gergely (2023b)
*Dicharax abdoui* Páll-Gergely, 2017	~ 9 km NE of Thakhek (Muang Khammouane), Khammouane Province, Laos (17°26.757'N, 104°52.937'E)	The type locality only	Páll-Gergely et al. (2017), Inkhavilay et al. (2019)
*Dicharax cristatus* (Möllendorff, 1886)	Hunan, China	Gansu, Shaanxi, Sichuan, Yunnan, Hunan, Hubei, Guangxi provinces, China; northern Vietnam; Luang Prabang Province, and Feuang District, Vientiane Province, Laos	Möllendorff (1886), Páll-Gergely et al. (2017), this study
*Dicharax depressus* (Bavay & Dautzenberg, 1912)	Pac-Kha, Tonkin [Pa Kha, Son La Province, Vietnam]	Northern Vietnam and northern Laos	Bavay and Dautzenberg (1912), Páll-Gergely et al. (2017), Inkhavilay et al. (2019)
*Dicharax feuangensis* Jirapatrasilp & Inkhavilay, sp. nov.	Unnamed limestone hill (MF1), Feuang District, Vientiane Province, Laos (18°40'29.2"N, 102°02'18.4"E)	The type locality only	This study
*Dicharax fimbriatus* (Bavay & Dautzenberg, 1912)	Pac-Kha, Tonkin [Pa Kha, Son La Province, Vietnam]	Guangxi, eastern Yunnan, Guizhou, northern Sichuan and southern Hunan, China; northern Vietnam; Luang Prabang Province, Laos	Bavay and Dautzenberg (1912), Páll-Gergely et al. (2017), Inkhavilay et al. (2019)
*Dicharax pallgergelyi* Jirapatrasilp & Inkhavilay, sp. nov.	Unnamed limestone hill (MF1), Feuang District, Vientiane Province, Laos (18°40'29.2"N, 102°02'18.4"E)	The type locality only	This study
*Dioryx bacca* (Pfeiffer, 1863)	Lao Mountains, Camboja [Laos or Cambodia]	Vientiane and Xayaboury provinces, Laos; Chiang Mai Province, Thailand	Pfeiffer (1863), Solem (1966), Inkhavilay et al. (2019)
*Dioryx cariniger* Möllendorff, 1897	Near Luang Phrabang, Laos	Luang Prabang and Khammouane provinces, Laos	Möllendorff (1897), Inkhavilay et al. (2019)
*Dioryx dongiensis* Varga, 1972	Cuc Phuong, Ninh Binh, Vietnam	The type locality and Feuang District, Vientiane Province, Laos (first record in Laos)	Varga (1972), this study
*Dioryx feuangensis* Jirapatrasilp & Inkhavilay, sp. nov.	Pha Thor, Feuang District, Vientiane Province, Laos (18°42'08.6"N, 102°07'54.5"E)	Feuang District, Vientiane Province, Laos	This study
*Dioryx messageri* (Bavay & Dautzenberg, 1900)	That-Khe [That Khe Town, Trang Dinh District, Lang Son Province, Vietnam]	Vientiane Province, Laos; northern Vietnam	Bavay and Dautzenberg (1900), Do et al. (2015), Inkhavilay et al. (2019)
*Metalycaeus heudei* (Bavay & Dautzenberg, 1900)	Haut Tonkin [Vietnam]	Yunnan, Hunan and Hubei provinces, China; Shan and Kayah States, Myanmar; northern Vietnam; Houaphanh and Luang Prabang provinces, Laos	Bavay and Dautzenberg (1900), Páll-Gergely et al. (2017, 2021), Inkhavilay et al. (2019), this study
*Metalycaeus laosensis* Páll-Gergely, 2017	Old forest near stream ~ 1 km SW of a stream and Nam Ou (river) confluence, Phongsaly Province, northern Laos (21°44.663'N, 102°10.999'E)	Phongsaly Province, Laos	Páll-Gergely et al. (2017), Inkhavilay et al. (2019)
*Pincerna costulosus* (Bavay & Dautzenberg, 1912)	Phong-Tho, Tonkin [Phong Tho District, Lai Chau Province, Vietnam]	Guangxi, China; Lai Chau and Lao Kay provinces, Vietnam; Phongsaly and Xiangkhoang provinces, Laos	Bavay and Dautzenberg (1912), Páll-Gergely (2023b)
*Pincerna mouhoti* (Pfeiffer, 1863)	Lao Mountains, Camboja [Laos or Cambodia]	Northern Vietnam and northern Laos	Pfeiffer (1863), Inkhavilay et al. (2019), Páll-Gergely (2023b)
*Pincerna panhai* Jirapatrasilp & Inkhavilay, sp. nov.	Unnamed limestone hill (MF1), Feuang District, Vientiane Province, Laos (18°40'29.2"N, 102°02'18.4"E)	Feuang District, Vientiane Province, Laos	This study
*Pincerna vanbuensis* (Bavay & Dautzenberg, 1900)	Van Bu, Tonkin [Son La Province, Vietnam]	Southern China, northern Vietnam; Luang Prabang, Luang Namtha, Khammouane, and Houaphanh provinces, Laos	Bavay and Dautzenberg (1900), Inkhavilay et al. (2019), Páll-Gergely (2023b), this study

Recent biodiversity surveys in the limestone-rich areas in Vientiane and Houaphanh provinces yielded shells from diverse taxa, some of which were already described as new to science (Inkhavilay et al. 2023, 2024; Inkhavilay and Sutcharit 2024; Sutcharit et al. 2025). Based on these shell collections, we discovered that some undetermined species from the Alycaeinae are conchologically distinct from other congeners from Laos, Thailand, and Vietnam (Páll-Gergely et al. 2017; Inkhavilay et al. 2019; Do and Nguyen 2022), and therefore, we describe them as new species below. In addition, the remaining specimens were identified as other nominal species belonging to either *Dicharax* Kobelt & Möllendorff, 1900, *Dioryx* Benson, 1859, *Metalycaeus* Pilsbry, 1900, or *Pincerna* Preston, 1907. These species are provided with morphological diagnoses, as well as a remark of their systematic status, and an updated list of all species from the Alycaeinae in Laos is also provided.

## Materials and methods

**Figure 1.**
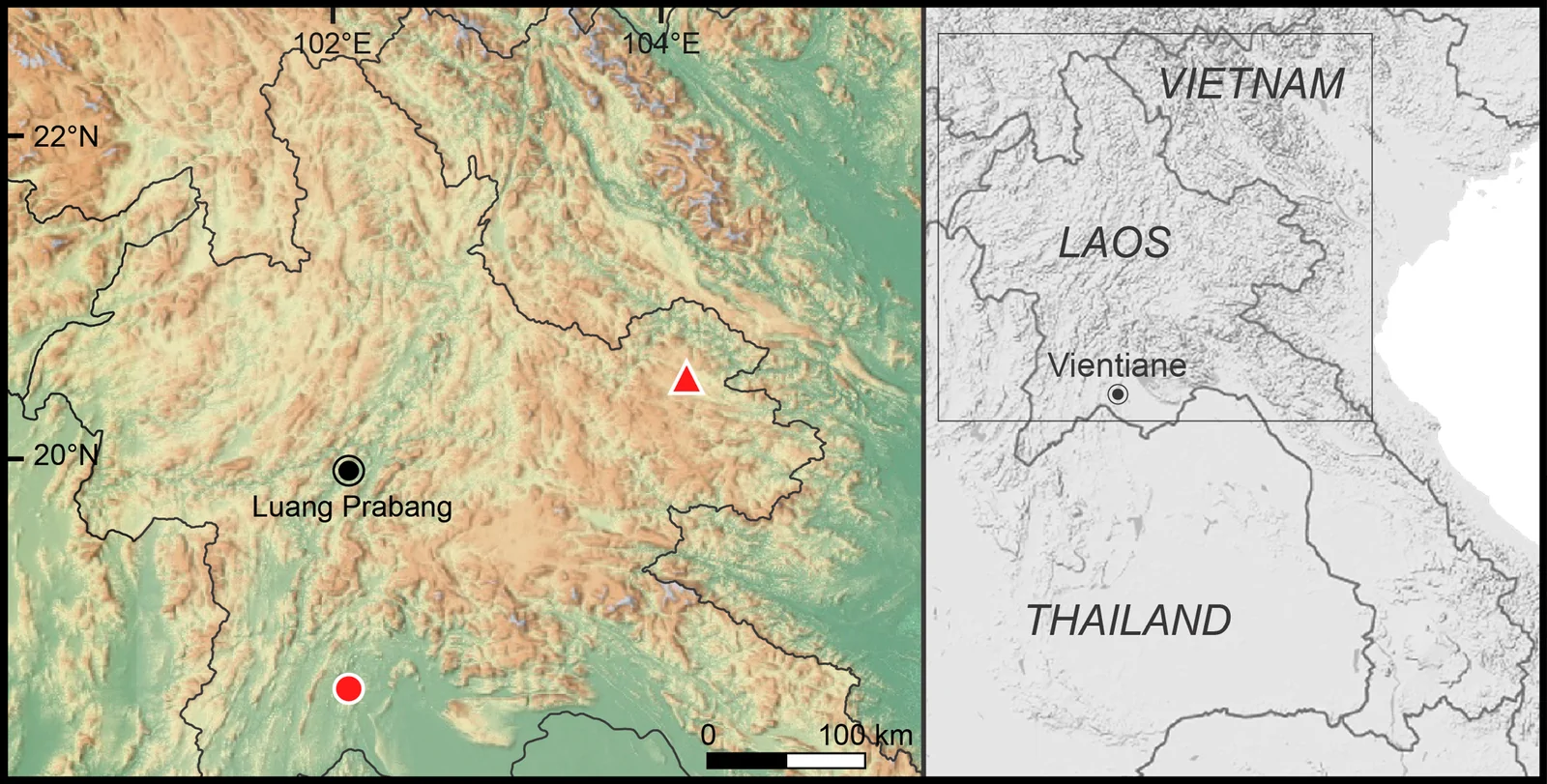
Geographical position of the limestone areas as collecting localities in Feuang District, Vientiane Province (red circle), and Viengxay District, Houaphanh Province (red triangle).

This study is based on material collected from Viengxay District, Houaphanh Province and Feuang District, Vientiane Province (Fig. 1), in 2022 and 2024, respectively. The fieldwork was conducted under a Memorandum of Agreement between the Center of Excellence in Biodiversity and Chulalongkorn University (CU, Thailand) and the National University of Laos (NUOL, Laos). Empty shells were searched and hand-collected from the forest floor around limestone outcrops, caves, and rock crevices at various sites in this area. Latitude, longitude, and elevation were obtained with a Garmin GPSMAP 60 CSx. Shells were photographed and measured under light microscopy using a Leica M205C microscope with a fusion optics stereo microscope and the Leica Application Suite Image System, and by scanning electron microscopy (SEM; JEOL, JSM-6610 LV) at Chulalongkorn University, Thailand. The counting of the shell whorls to the nearest quarter follows Kerney and Cameron (1979). The nomenclature of external shell features of the Alycaeinae and the three-part division of the teleoconch, namely R1 (the beginning of the teleoconch to the beginning of the differently ribbed region along the suture), R2 (the differently ribbed area to the constriction), and R3 (the constriction to the peristome), follow Páll-Gergely et al. (2017) and Jirapatrasilp et al. (2021).

The shells were initially identified and classified into their respective genera following Páll-Gergely et al. (2017, 2020d, 2021, 2024), and Jirapatrasilp et al. (2021), and then compared with some relevant type specimens and images of authenticated specimens.

### Institutional abbreviations

**CUMZ**, Chulalongkorn University Museum of Zoology, Bangkok; **NHM**, The Natural History Museum, London; **NHMUK**, when citing specimen lots deposited in the NHM; **SMF**, Forschungsinstitut und Naturmuseum Senckenberg, Frankfurt am Main.

### Other abbreviations

**SH**, shell height; **SW**, shell width.

### Taxon names

Descriptions of new species in this study are attributed to the first and second authors. The complete citation of the new species is Jirapatrasilp & Inkhavilay in Jirapatrasilp et al.

## Results

A total of eight species from the subfamily Alycaeinae were reported in this study. Among these, four species are new to science and described herein based on their distinct shell morphology. They are *Dioryx feuangensis* Jirapatrasilp & Inkhavilay, sp. nov., *Dicharax feuangensis* Jirapatrasilp & Inkhavilay, sp. nov., *Dicharax pallgergelyi* Jirapatrasilp & Inkhavilay, sp. nov., and *Pincerna panhai* Jirapatrasilp & Inkhavilay, sp. nov. The remaining four nominal species are *Metalycaeus heudei* (Bavay & Dautzenberg, 1900), *Dioryx dongiensis* Varga, 1972, *Dicharax cristatus* (Möllendorff, 1886), and *Pincerna vanbuensis* (Bavay & Dautzenberg, 1900). One species, *Dioryx dongiensis* is recorded from Laos for the first time.

### Taxonomy and systematics

**Family Cyclophoridae Gray, 1847**

**Subfamily Alycaeinae Blanford, 1864**

#### Metalycaeus

Taxon classificationAnimaliaArchitaenioglossaAlycaeidae

Pilsbry, 1900

8F03BD78-4280-5C2A-8AD4-625CA6266A97

Alycæus (Metalycæus) Pilsbry, 1900: 382.
Metalycaeus – Páll-Gergely et al. 2020d: 137–138. Páll-Gergely et al. 2021: 45. Gittenberger et al. 2024: 190.

##### Type species.

Alycaeus (Metalycaeus) melanopoma Pilsbry, 1900 (synonym of *Alycaeus nipponensis* Reinhardt, 1877, see Minato 1988), by subsequent designation (Thiele 1929).

##### Remarks.

This genus is diagnosed by the presence of spiral striation on both the protoconch and teleoconch. However, there is at least one species, *M. pygmachos* Páll-Gergely & Hunyadi, 2021 from Myanmar, lacking spiral striation on the entire shell, but this species is assigned to *Metalycaeus* based on its large size and the depressed swelling on R3. Therefore, the lack of spiral striation on the protoconch is deemed a secondary reduction, and it is necessary to consider other shell characters in order to classify the specimens into *Metalycaeus*. At present, there are 70 species of *Metalycaeus* known from southeastern Himalaya to the south of Honshu Island, Japan, the northern islands of the Philippines, northern Laos and Vietnam, and central Thailand (Páll-Gergely et al. 2020d, 2021; Gittenberger et al. 2024). See earlier taxonomic treatment, extended diagnosis, and other taxonomic remarks of *Metalycaeus* in Páll-Gergely et al. (2020d) and Gittenberger et al. (2024).

#### Metalycaeus heudei

Taxon classificationAnimaliaArchitaenioglossaAlycaeidae

(Bavay & Dautzenberg, 1900)

521FD8D6-E33D-53B6-9A75-71C45778F2EE

Figs 2A, 3

Alycaeus (Charax) heudei Bavay & Dautzenberg, 1900: 121–122. Type locality: Haut-Tonkin.
Metalycaeus heudei – Páll-Gergely et al. 2020d: 143–144. Páll-Gergely et al. 2021: 53, fig. 41.

##### Material examined.

**Figure 2.**
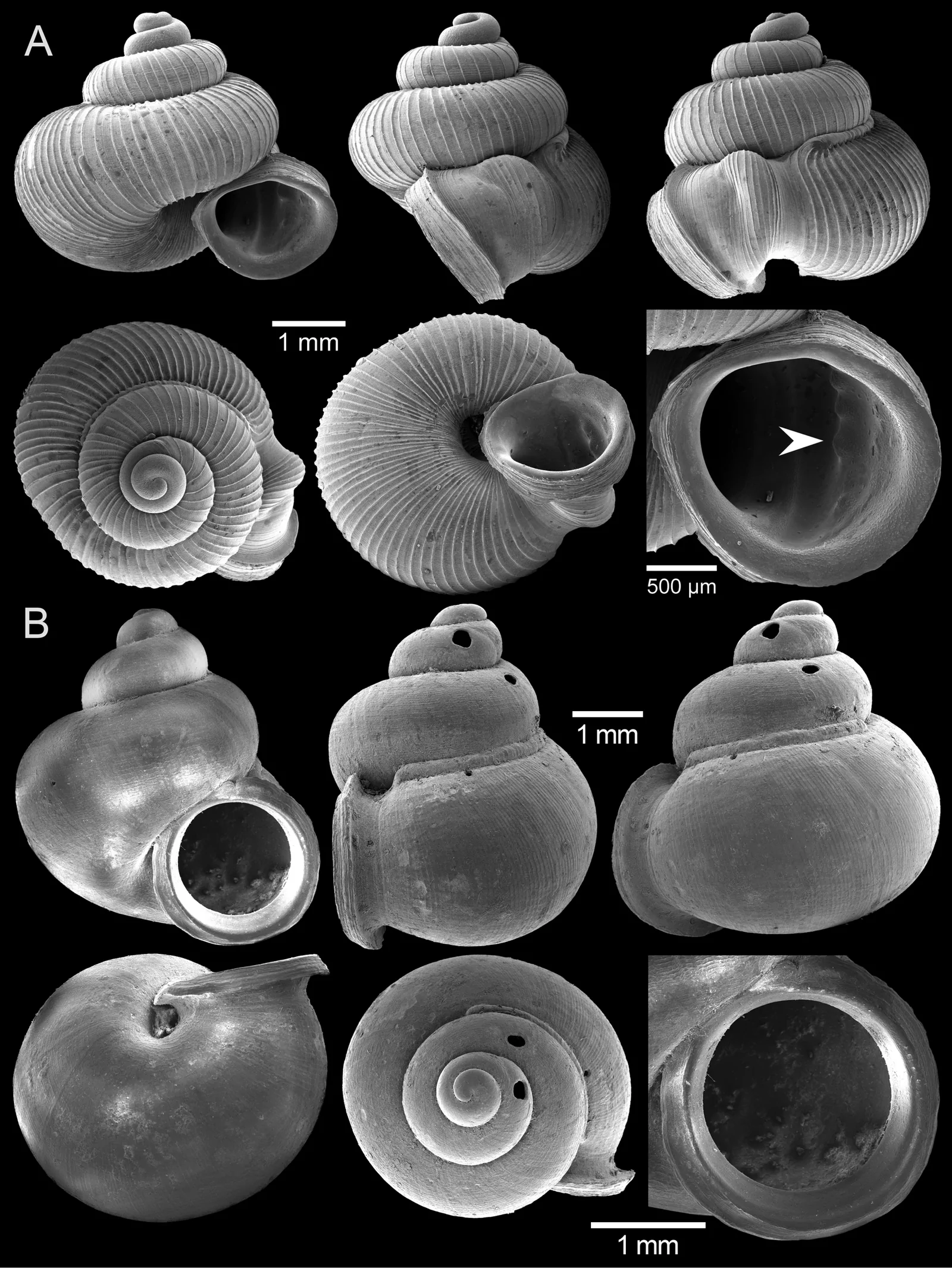
SEM images of **A**. *Metalycaeus heudei*, specimen CUMZ 14473, and **B**. *Dioryx dongiensis*, specimen CUMZ 14476, in different positions of the whole shell, and a close-up image of the aperture. The white arrow points to the wavy ridge inside the apertural tube.

**Figure 3.**
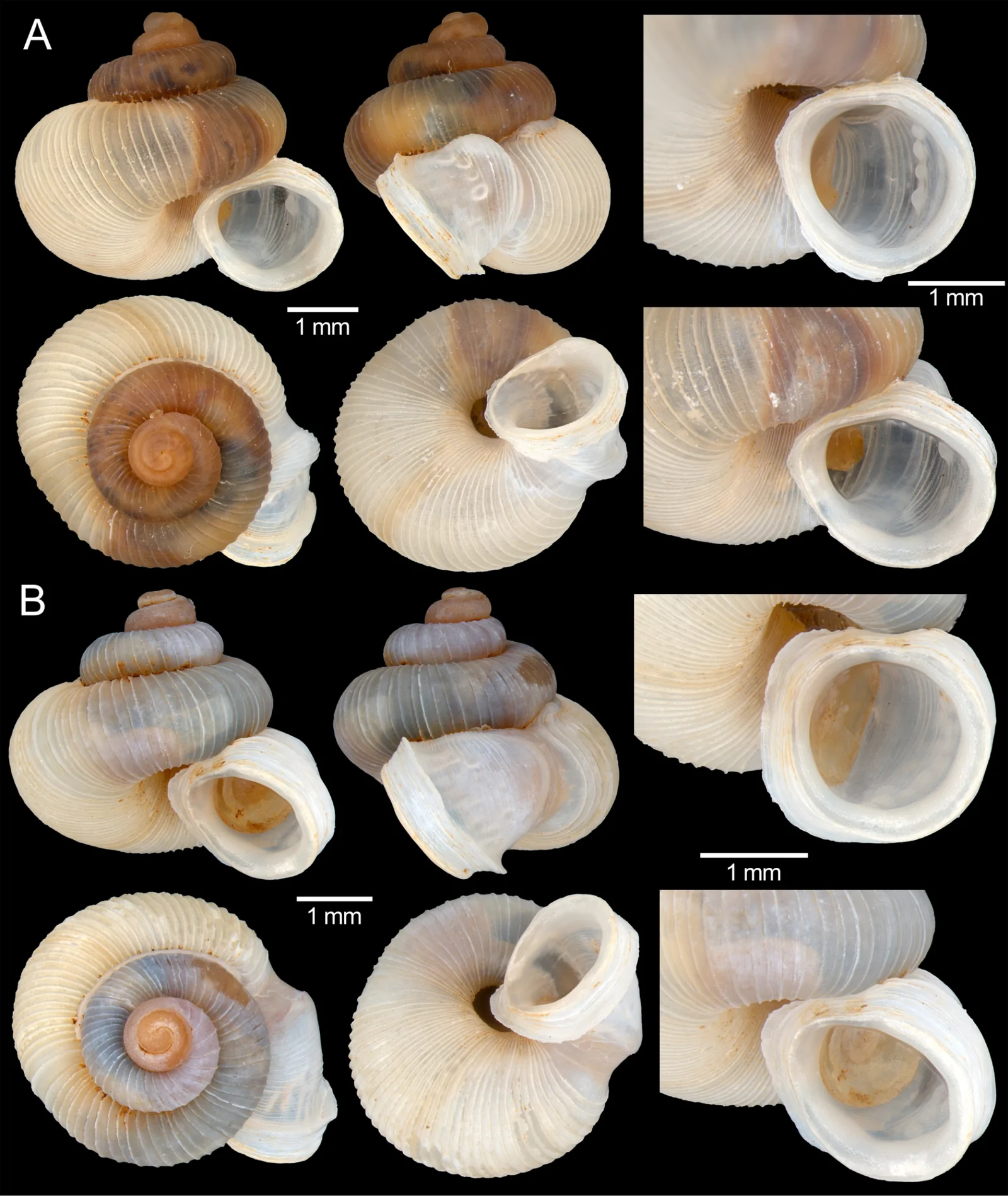
*Metalycaeus heudei* in different positions of the whole shell, and a close-up image of the aperture. Specimens **A**. CUMZ 14473, and **B**. CUMZ 14474.

Laos • 2 shells; Houaphanh Province, Viengxay District, Ban Nathan village; 20°27'28.1"N, 104°08'43.4"E; 965 m a.s.l.; 6 Oct. 2022; leg. K. Inkhavilay; CUMZ 14473–14474 (Figs 2A, 3) • 17 shells; Houaphanh Province, Viengxay District, Ban Hang Long village; 20°27'38.7"N, 104°10'28.6"E; 840 m a.s.l.; 6 Oct. 2022; leg. K. Inkhavilay; CUMZ 14475.

##### Diagnosis.

Shell depressed to conical. Protoconch scaly; spiral striation starting from R1, continuing to the end of R2. R1 ~ 1¾ whorls with regular, strong radial ribs. R2 ~ ¼ whorl and nearly twice as long as R3; with ~ 32 ribs. R3 with less distinct ribs, strongly coiling downwards being highly oblique to horizontal axis of the shell, slightly expanding near the end of the aperture. Inner peristome blunt, slightly protruding. Outer peristome thickened, multilayered, expanded in various degrees, not reflected. Apertural lip finely granulated, mostly with 4–6 wavy ridges inside the apertural tube at the position when the tube starting to coil downward. Umbilicus round, close.

##### Differential diagnosis.

See Páll-Gergely et al. (2017: 83).

##### Distribution.

Yunnan, Hunan and Hubei provinces, China; Shan and Kayah States, Myanmar; northern Vietnam; Luang Prabang (Páll-Gergely et al. 2017, 2021) and Houaphanh provinces, Laos.

##### Remarks.

*Metalycaeus heudei* is one of the most widely distributed alycaein species known, with high variation in shell size, shell shape from depressed to conical, R2 length and shape (bulging or not), and the strength of the swelling on R3. The shells from Houaphanh Province, Laos in this study correspond to the conical end of the shell shape spectrum (*paviei* form). These shells are also very similar to the type material of Alycaeus paviei var. minor Bavay & Dautzenberg, 1912 in having a close umbilicus and ridges inside the apertural tube at the position when the tube starting to coil downward. In addition, a lack of spiral striation on the protoconch was detected in these shells, aligning to the previous report that not all shells from the same *Metalycaeus* species possess this character (Páll-Gergely et al. 2017, 2021). See earlier taxonomic treatment of *M. heudei* in Páll-Gergely et al. (2021).

#### Dioryx

Taxon classificationAnimaliaArchitaenioglossaAlycaeidae

Benson, 1859

A9F0F327-D8D8-5449-BB83-04EF204C367D

Alycæus (Dioryx) Benson, 1859: 177.
Dioryx – Páll-Gergely et al. 2020d: 121–122.

##### Type species.

*Alycaeus amphora* Benson, 1856, by subsequent designation (Gude 1921: 198).

##### Remarks.

This genus is diagnosed by a globular or high-spired shell, sometimes the body whorl is angled or keeled, a smooth protoconch, a reduced sculpture in teleoconch, and an absence of R3. At present, there are 27 species of *Dioryx* known from the southeastern Himalayan region to Taiwan in the east, and down to the northern part of the Malay Peninsula and southern Vietnam in the south (Páll-Gergely et al. 2020d). See earlier taxonomic treatment, extended diagnosis, and other taxonomic remarks of *Dioryx* in Páll-Gergely et al. (2020d).

#### Dioryx dongiensis

Taxon classificationAnimaliaArchitaenioglossaAlycaeidae

Varga, 1972

92E5B232-5A76-571C-8643-60157360FD8F

Fig. 2B

Dioryx dongiensis Varga, 1972: 135, 137, figs 21–23. Type locality: Vietnam, Tonkin, Cuc Phuong bei Dong [Cuc Phuong, Ninh Binh]. Páll-Gergely et al. 2020d: 125. von Oheimb et al. 2025: tables 1, 3, 4, fig. 8h.

##### Material examined.

Laos • 1 shell; Vientiane Province, Feuang District, limestone hill behind Xinxayyaram Temple (loc. MF10); 18°33'00.5"N, 101°58'30.8"E; 265 m a.s.l.; 9 Oct. 2024; leg. CU-NUOL expedition team; CUMZ 14476 (Fig. 2B).

##### Diagnosis.

Shell conical, globular. Spire ~ 30% of shell height. Protoconch smooth. R1 ~ 3½ whorls, sculptured with indistinct spiral striation. R2 ~ ¼ whorl, with thin ribs; the end of R2 slightly narrower. Peristome double with expanding outer peristome; totally adnate to the last whorl. Umbilicus slightly open.

##### Differential diagnosis.

*Dioryx dongiensis* is different from *D. kobeltianus* (Möllendorff, 1874) in having a shallower umbilicus with a less pronounced edge of the last whorl’s lower periphery. In addition, the peristome of *D. kobeltianus* is not totally adnate to the last whorl (Páll-Gergely et al. 2017).

##### Distribution.

The type locality (von Oheimb et al. 2025), and Feuang District, Vientiane Province, Laos.

##### Remarks.

This is the first record of *Dioryx dongiensis* in Laos, apart from the type locality in Vietnam (von Oheimb et al. 2025). Although the locality in Laos is more than 400 km away from the type locality, this shell is herein identified to this species based on its shell shape and the peristome totally adnate to the last whorl. Moreover, several Alycaeinae species have a wide distribution from Vietnam to Laos (e.g., *Dicharax depressus*; Páll-Gergely et al. 2017). As the shell of this species is very small, soil sieving and floatation methods (Liew et al. 2008) are preferred to retrieve more shells, and this species might be collected from more localities located between Vietnam and Laos. See earlier taxonomic treatment of *Dioryx dongiensis* in Páll-Gergely et al. (2020d).

#### Dioryx feuangensis

Taxon classificationAnimaliaArchitaenioglossaAlycaeidae

Jirapatrasilp & Inkhavilay sp. nov.

30AF2FFE-C95D-56EA-86C3-787C7ED13CA5

https://zoobank.org/42B60A70-0ED1-455B-87AC-0732CCB6DB6F

Fig. 4

##### Type material.

**Figure 4.**
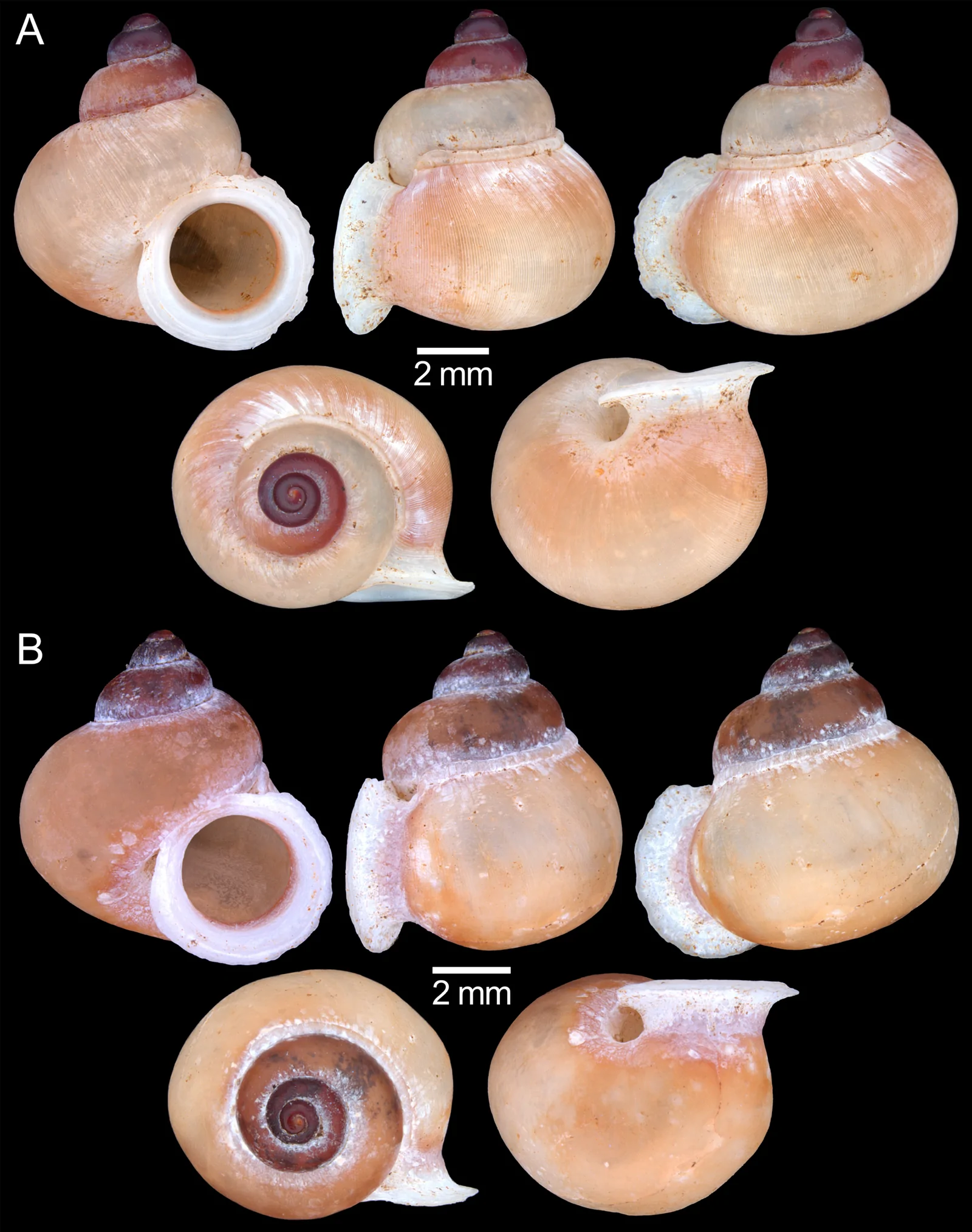
*Dioryx feuangensis* sp. nov. in different positions of the whole shell. **A**. Holotype CUMZ 14477, and **B**. Specimen CUMZ 14479.

***Holotype***. Laos • 1 shell, SH = 9.3 mm, SW = 8.3 mm; Vientiane Province, Feuang District, Pha Thor (loc. MF13); 18°42'08.6"N, 102°07'54.5"E; 225 m a.s.l.; 10 Oct. 2024; leg. CU-NUOL expedition team; CUMZ 14477 (Fig. 4A). ***Paratypes***. Laos • 33 shells; same data as for holotype; CUMZ 14478 • 3 shells; same data as for holotype; NHMUK 20260369 • 3 shells; same data as for holotype; SMF.

##### Other material.

Laos • 224 shells; Vientiane Province, Feuang District, Pha Reung (loc. MF14); 18°44'20.4"N, 102°07'02.9"E; 360 m a.s.l.; 10 Oct. 2024; leg. CU-NUOL expedition team; CUMZ 14479 (Fig. 4B).

##### Diagnosis.

Shell conical, globular. Spire ~ ¼ of shell height. Spiral striation lacking on the entire shell. R1 ~ 2½ whorls; R2 ~ ½ whorl, with indistinct ribs; the end of R2 narrower, appearing as a broad constriction between R2 and apertural tube. Peristome double with prominent, expanding outer peristome with irregular perimeter. Umbilicus open.

##### Differential diagnosis.

*Dioryx feuangensis* sp. nov. is distinct in having a prominent, expanding outer peristome with irregular perimeter. Apart from this character, this new species is similar to *D. compactus* (Bavay & Dautzenberg, 1900) (Páll-Gergely et al. 2017: fig. 4c) in its globular shell shape with a SW:SH ratio approaching 1, but the new species differs by a larger shell (SH 8.5–9.5 mm, SW 8.0–8.5 mm vs SH 4.7 mm, SW 4.2 mm) with matt surface. This new species is also similar to *D. rosea* (Bavay & Dautzenberg, 1900) in its shell colour and shape, but the new species differs by a lack of spiral striation on the entire shell, and a less protruding inner peristome. The new species’ matt shell surface matches more to *D. globuloides* Zilch, 1957 (Páll-Gergely et al. 2017: fig. 4d), but the latter species is smaller, with a shorter sutural tube at R2, and the outer peristome is expanded to a lesser extent. *Dioryx feuangensis* sp. nov. is similar to *D. requiescens* (Mabille, 1887) in its R2 length, but *D. feuangensis* sp. nov. is more globular in shell shape, and has an outer peristome with irregular perimeter, and an apertural tube opening parallel to the horizontal axis of the shell. *Dioryx requiescens* is less globular (SW:SH ratio < 1), and has a regular outer peristome, and an apertural tube opening obliquely downward to the horizontal axis of the shell.

##### Description.

Shell conical, globular, solid, matt, yellowish to pale crimson, SH 8.5–9.5 mm, SW 8.0–8.5 mm (*n* = 10). Shell outline round in apical view, spire ~ ¼ of shell height. Whorls ~ 4½, last whorl large, round; spiral striation lacking on the entire shell. Protoconch low, ~ 1½ whorls, glossy, smooth. R1 ~ 2½ whorls; R2 ~ ½ whorl, with indistinct ribs; the end of R2 narrower, appearing as a broad constriction between R2 and apertural tube. R3 absent; apertural tube expanding from the end of R2, opening parallel to horizontal axis of the shell. Aperture round; peristome double with prominent outer peristome with irregular perimeter. Inner peristome blunt, slightly protruding. Outer peristome not thickened, expanded, not reflected. Umbilicus open. Operculum unknown.

##### Etymology.

The species name is derived from the type locality, Feuang District in Vientiane Province, Laos.

##### Distribution.

Known only from the limestone areas in Feuang District, Vientiane Province, Laos.

##### Remarks.

The lack of spiral striation on the entire shell and a prominent, expanding outer peristome with irregular perimeter are consistent across all specimens of this new species, suggesting that these are important diagnostic characters.

#### Dicharax

Taxon classificationAnimaliaArchitaenioglossaAlycaeidae

Kobelt & Möllendorff, 1900

2AD2885A-2C1E-50E6-9FDD-189701DAB90B

Alycaeus (Dicharax) Kobelt & Möllendorff, 1900: 186 (replacement name for Charax Benson, 1859, non Charax Scopoli, 1777 [Pisces]).
Dicharax – Jirapatrasilp et al. 2021: 4. Páll-Gergely et al. 2021: 21. Gittenberger et al. 2024: 199. Matsuda and Yano 2024: 3. Páll-Gergely et al. 2025: 50.

##### Type species.

*Alycaeus hebes* Benson, 1857, by subsequent designation (Gude 1921: 236).

##### Remarks.

This genus is diagnosed by a low protoconch, and the entire shell lacking spiral striation. This genus comprises nearly 200 species and is widely distributed from the Western Ghats of India and in the southwestern and southeastern Himalayan region to Japan, through the Malay Peninsula to the southern arc of the Malay Archipelago up to Sumatra and Java (Páll-Gergely et al. 2020d). See earlier taxonomic treatment, extended diagnosis, and other taxonomic remarks of *Dicharax* in Jirapatrasilp et al. (2021).

#### Dicharax cristatus

Taxon classificationAnimaliaArchitaenioglossaAlycaeidae

(Möllendorff, 1886)

7E9E5638-6434-5A15-8785-1F6D0D763C51

Figs 5A, 6

Alycaeus cristatus Möllendorff, 1886: 168, pl. 5, fig. 6. Type locality: Hunan, China.
Dicharax cristatus – Páll-Gergely et al. 2017: 34–43, figs 12c, d, 13c, 24–27, 28a–d, 29c, d, 30. Páll-Gergely et al. 2020d: 57.

##### Material examined.

**Figure 5.**
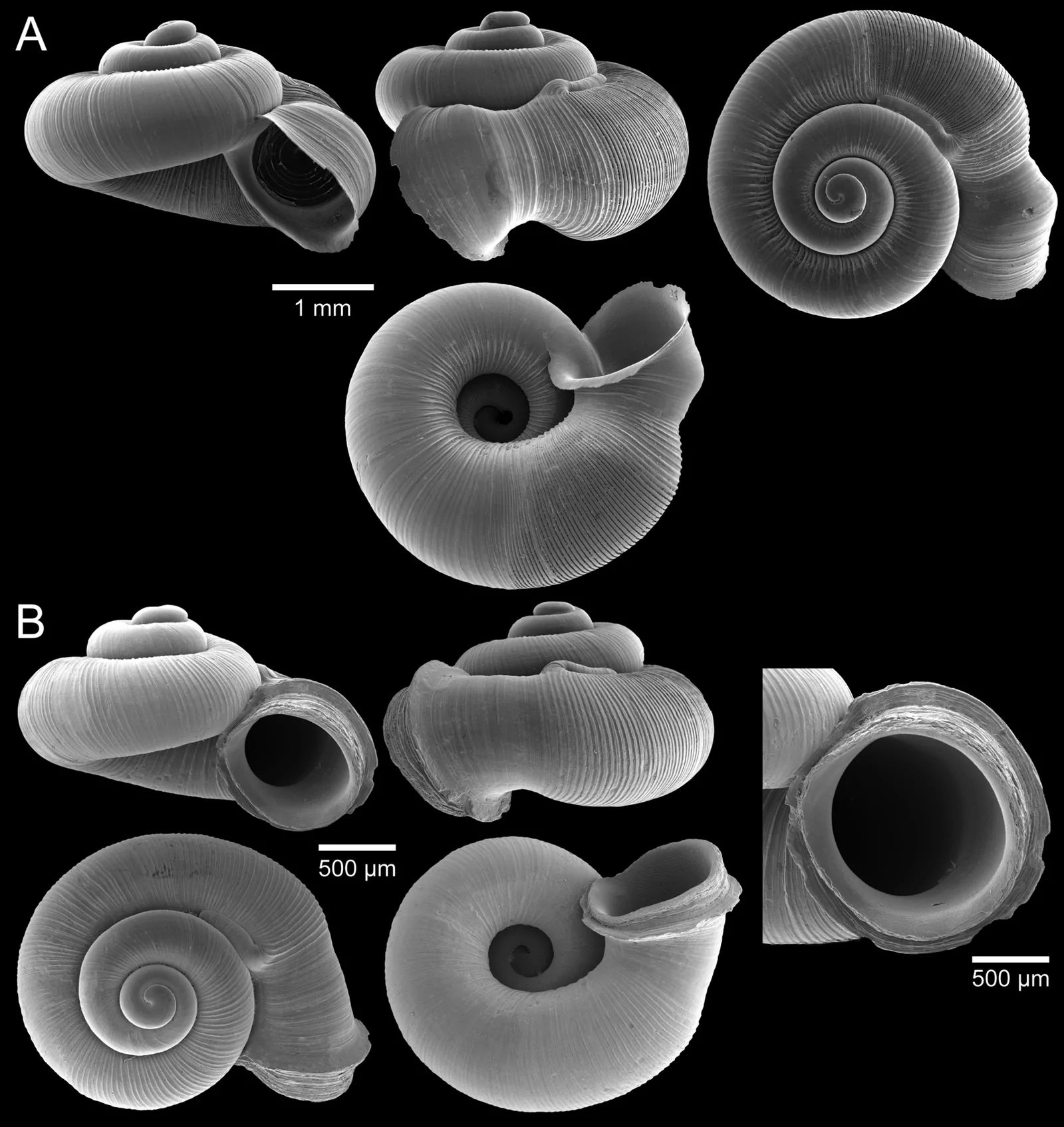
SEM images of **A**. *Dicharax cristatus*, CUMZ 14480, and **B**. *Dicharax pallgergelyi* sp. nov., holotype CUMZ 14482, in different positions of the whole shell and a close-up image of the aperture.

**Figure 6.**
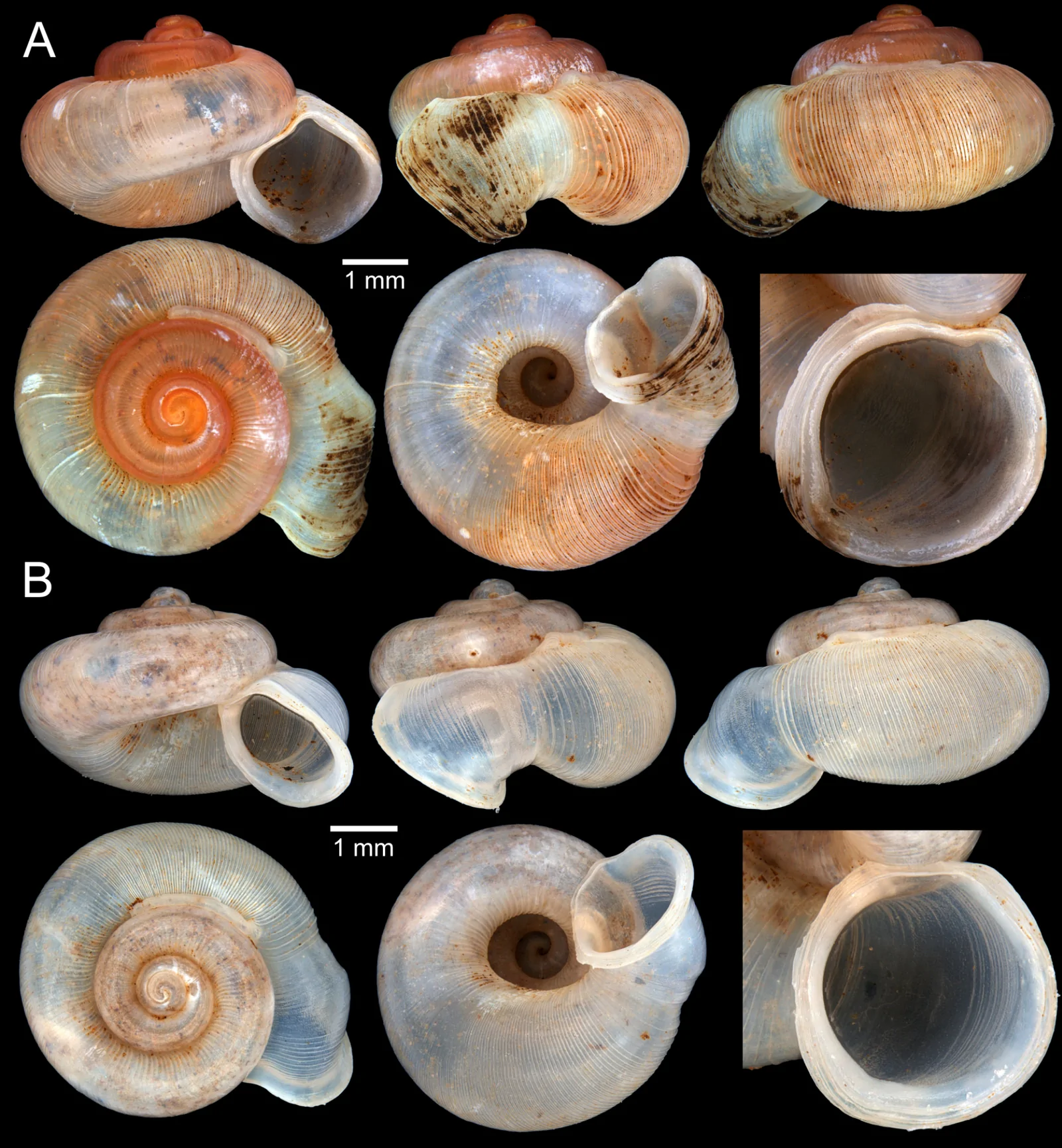
*Dicharax cristatus*, specimens CUMZ 14480 from the same collecting locality as of Fig. 5A, in different positions of the whole shell and a close-up image of the aperture.

Laos • 139 shells; Vientiane Province, Feuang District, unnamed limestone hill (loc. MF1); 18°40'29.2"N, 102°02'18.4"E; 320 m a.s.l.; 7 Oct. 2024; leg. CU-NUOL expedition team; CUMZ 14480 (Figs 5A, 6) • 34 shells; Vientiane Province, Feuang District, Pha Reung (loc. MF14); 18°44'20.4"N, 102°07'02.9"E; 360 m a.s.l.; 10 Oct. 2024; leg. CU-NUOL expedition team; CUMZ 14481.

##### Diagnosis.

Shell depressed-conical. Spire ~ 1/5 of shell height. R1 with regular ribs; R2 as long as or slightly shorter than R3. R3 with sharp or moderately sharp swelling, in the middle of R3 or close to the aperture. Inner peristome sometimes with conspicuous upper incision. Outer peristome expanded, not reflected.

##### Differential diagnosis.

See Páll-Gergely et al. (2017: 43).

##### Distribution.

Gansu, Shaanxi, Sichuan, Yunnan, Hunan, Hubei, Guangxi provinces, China; northern Vietnam; Luang Prabang Province (Páll-Gergely et al. 2017), and Feuang District, Vientiane Province, Laos.

##### Remarks.

The current species boundary of *D. cristatus* based on morphological characters is wide, varying in shell size, spire height (flat to somewhat elevated), peristome (strongly crenulated to not crenulated), R1 sculpture (smooth to regularly ribbed), and the absence or presence or the position of the swelling on R3. Therefore, there are no unique set of characters unequivocally defining *D. cristatus* (Páll-Gergely et al. 2017). It is possible that future molecular studies may show that this nominal taxon contains multiple lineages. See earlier taxonomic treatment of *D. cristatus* in Páll-Gergely et al. (2017, 2020d).

#### Dicharax pallgergelyi

Taxon classificationAnimaliaArchitaenioglossaAlycaeidae

Jirapatrasilp & Inkhavilay sp. nov.

9FDE9B76-1CE9-5B2B-9ACC-D60A5FBFCA72

https://zoobank.org/A845948E-8AA6-430F-804B-3B217B07557A

Figs 5B, 7

##### Type material.

**Figure 7.**
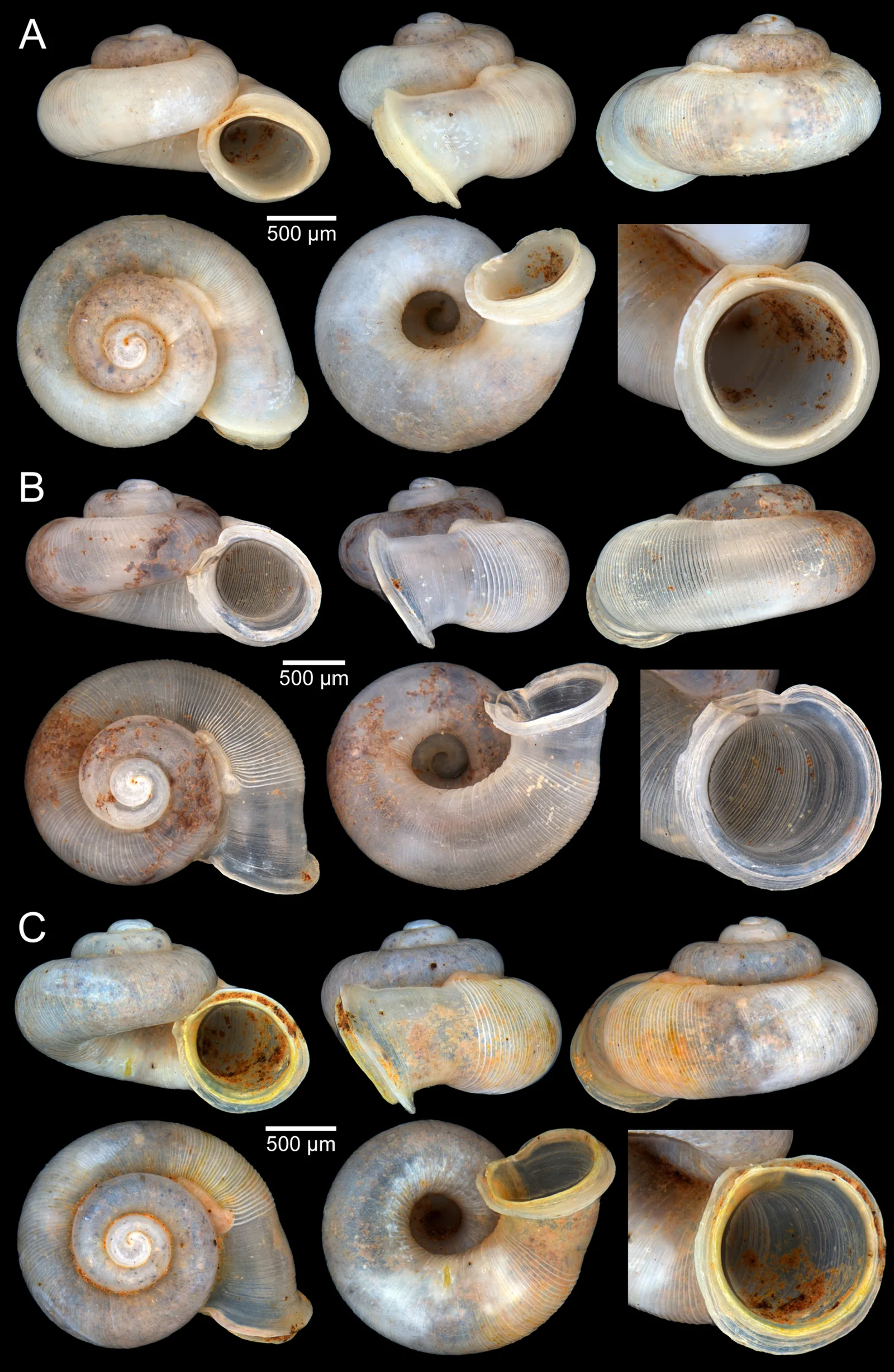
*Dicharax pallgergelyi* sp. nov., paratypes CUMZ 14482A in different positions of the whole shell and a close-up image of the aperture.

***Holotype***. Laos • 1 shell, SH = 1.5 mm, SW = 2.3 mm; Vientiane Province, Feuang District, unnamed limestone hill (loc. MF1); 18°40'29.2"N, 102°02'18.4"E; 320 m a.s.l.; 7 Oct. 2024; leg. CU-NUOL expedition team; CUMZ 14482 (Fig. 5B). ***Paratypes***. Laos • 5 shells; same data as for holotype; CUMZ 14482A (Fig. 7).

##### Diagnosis.

Shell depressed-conical. Spire ~ 1/5 of shell height. R1 ~ 1¾ whorls with regular, very low ribs. R2 ~ 1/5 whorl and nearly as long as or slightly shorter than R3; with ~ 25 ribs; ribs becoming thicker at the end of R2. R3 ~ 1/5 whorl with very fine, close-set ribs. Apertural tube coiling slightly downward from the horizontal axis of the shell, expanding near the end of the aperture. Inner peristome blunt, thick, protruding. Outer peristome thickened, multilayered, expanded, not reflected.

##### Differential diagnosis.

*Dicharax pallgergelyi* sp. nov. is similar to *D. notus* (Godwin-Austen, 1914) (Páll-Gergely et al. 2025: fig. 71a–e) in shell shape and size, but the new species possesses a lesser number of R2 ribs (25–28) with lower rib height, compared to those in *D. notus* (48–50, more elevated R2 ribs with T-shaped cross section). In addition, *D. pallgergelyi* sp. nov. also differs by a distinct protruding inner peristome which is blunt and thick.

##### Description.

Shell depressed-conical, solid, translucent, SH 1.4–1.5 mm, SW 2.1–2.4 mm (*n* = 6). Shell outline oval in apical view, spire ~ 1/5 of shell height. Whorls ~ 3¾; spiral striation lacking on the entire shell. Protoconch low, ~ 1¾ whorls, smooth. Radial ribs regular and very low, indistinct starting from R1, becoming more distinct toward the end of R2, and finer, more close-set, and less distinct in R3. R1 ~ 1¾ whorls with ~ 38 ribs in ¼ whorl adjoining R2. Boundary between R1 and R2 distinct as R2 contains thicker and more distinct ribs. R2 ~ 1/5 whorl and nearly as long as or slightly shorter than R3; with ~ 25 ribs; ribs becoming thicker at the end of R2. Boundary between R2 and R3 distinct as a slight constriction. R3 ~ 1/5 whorl with very fine, close-set ribs; apertural tube coiling slightly downward from the horizontal axis of the shell, expanding near the end of the aperture. Aperture round. Peristome double with prominent outer peristome. Inner peristome blunt, thick, protruding. Outer peristome thickened, multilayered (visible in lateral view), expanded, not reflected. Umbilicus elliptical, open, approximately one third of shell width. Operculum unknown.

##### Etymology.

The species name is in honour of Dr. Barna Páll-Gergely, a prominent Hungarian malacologist, who extensively studied the taxonomy of the Alycaeinae and first recognised this species as new to science.

##### Distribution.

Known only from the type locality in Feuang District, Vientiane Province, Laos.

##### Remarks.

The number range of R2 ribs and the occurrence of the protruding inner peristome are consistent across all specimens of this new species, suggesting that these are important diagnostic characters.

#### Dicharax feuangensis

Taxon classificationAnimaliaArchitaenioglossaAlycaeidae

Jirapatrasilp & Inkhavilay sp. nov.

7843E635-DF5B-5518-9279-0FADDA8BB80B

https://zoobank.org/3A9476ED-09CC-456E-8025-54ABA193E503

Fig. 8

##### Type material.

**Figure 8.**
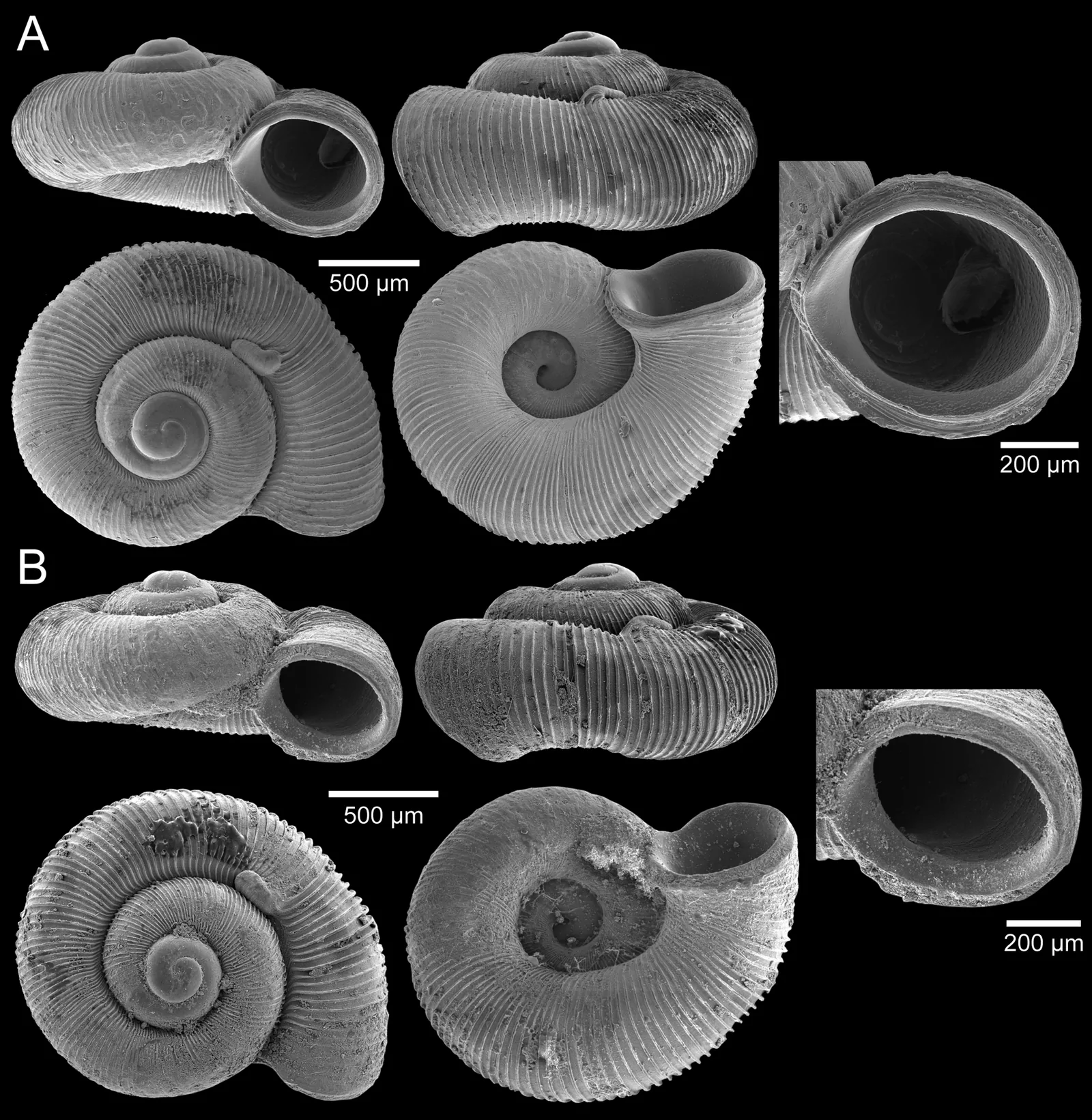
SEM images of *Dicharax feuangensis* sp. nov. in different positions of the whole shell and a close-up image of the aperture. **A**. Holotype CUMZ 14483; **B**. Paratype CUMZ 14484.

***Holotype***. Laos • 1 shell. SH = 1.0 mm, SW = 1.9 mm; Vientiane Province, Feuang District, unnamed limestone hill (loc. MF1); 18°40'29.2"N, 102°02'18.4"E; 320 m a.s.l.; 7 Oct. 2024; leg. CU-NUOL expedition team; CUMZ 14483 (Fig. 8A). ***Paratypes***. Laos • 1 shell; same data as for holotype; CUMZ 14484 (Fig. 8B).

##### Diagnosis.

Shell lenticular, depressed-conical. Spire ~ 1/5 of shell height. R1 ~ 1½ whorls with regular, sharp radial ribs. R2 very short; with ~ 10 ribs; ribs consistently thickened throughout. R3 ~ 1/5 whorl with ~ 21 ribs. Apertural tube coiling parallel to horizontal axis of the shell, slightly expanding near the end of the aperture. Inner peristome slim, very slightly protruding. Outer peristome thickened, slightly expanded at the columellar corner, not reflected.

##### Differential diagnosis.

Among the tiny *Dicharax* species (SH and SW < 2 mm), *D. feuangensis* sp. nov. is most similar to *D. parvulus* (Möllendorff, 1887) (Páll-Gergely et al. 2021: fig. 31b) in its low spire, short R2, and a nearly consistent radial rib density along the teleoconch, rendering the boundaries between R1 and R2, and R2 and R3 indistinct. However, the new species has a longer R3 with more ribs (21 vs 14), a less widening R3, and an outer peristome with only a slight expansion at the columellar corner. This new species is also similar to *D. abdoui* Páll-Gergely, 2017 (Páll-Gergely et al. 2017: fig. 6) in R2 length, but *D. feuangensis* sp. nov. has distinct higher elevated radial ribs throughout R1–R3, and a smooth protoconch, while *D. abdoui* has nearly smooth R1 and R3, and a protoconch with extremely fine pits arranged in spiral rows.

##### Description.

Shell lenticular, depressed-conical, solid, translucent, SH 1.0 mm, SW 1.9 mm (*n* = 2). Shell outline oval in apical view, spire ~ 1/5 of shell height. Whorls ~ 3½; spiral striation lacking on the entire shell. Protoconch low, ~ 1¾ whorls, smooth. Radial ribs strong, regular, starting from R1, becoming sharper, thicker, and less close-set toward the end of aperture. R1 ~ 1½ whorls with ~ 33 ribs in ¼ whorl adjoining R2. Boundary between R1 and R2 indistinct as R2 contains nearly the same rib density and thickness as in R1. R2 very short; with ~ 10 ribs; ribs consistently thickened throughout. Boundary between R2 and R3 indistinct as R3 contains nearly the same rib density and thickness as in R2. R3 ~ 1/5 whorl with ~ 21 ribs; apertural tube coiling parallel to horizontal axis of the shell, slightly expanding near the end of the aperture. Aperture round. Peristome double with regular outer peristome. Inner peristome slim, slightly protruding. Outer peristome thickened, multilayered (visible in lateral view), slightly expanded at the columellar corner, not reflected. Umbilicus elliptical, open, approximately 1/3 of shell width. Operculum thin, translucent; multispiral ridges not elevated.

##### Etymology.

The species name is derived from the type locality, Feuang District in Vientiane Province, Laos.

##### Distribution.

Known only from the type locality in Feuang District, Vientiane Province, Laos.

##### Remarks.

The length and the less widening of R3, and a smooth protoconch are consistent across all specimens of this new species, suggesting that these are important diagnostic characters.

#### Pincerna

Taxon classificationAnimaliaArchitaenioglossaAlycaeidae

Preston, 1907

4E19A43A-89F0-5978-81FF-4A9DE04FD1E0

Alycæus (Pincerna) Preston, 1907: 206.
Pincerna – Do and Nguyen 2022: 365. Páll-Gergely 2023b: 259.

##### Type species.

Alycæus (Pincerna) liratula Preston, 1907, by original designation.

##### Remarks.

This genus is diagnosed by a high spired, globose triangular shell, a smooth protoconch, a very short to short R2, and a closed umbilicus. At present, there is a total of 13 species of *Pincerna* known from northern Vietnam and northern Laos, and Sumatra, the southern part of the Malay Peninsula, and northern Borneo (Páll-Gergely et al. 2020d; Gittenberger et al. 2022). See earlier taxonomic treatment, extended diagnosis and other taxonomic remarks of *Pincerna* in Do and Nguyen (2022) and Páll-Gergely et al. (2024).

#### Pincerna vanbuensis

Taxon classificationAnimaliaArchitaenioglossaAlycaeidae

(Bavay & Dautzenberg, 1900)

54BDA813-75B6-5412-AFB6-A3B52425C691

Fig. 9

Alycaeus (*Dyorix* [sic]) vanbuensis Bavay & Dautzenberg, 1900: 120. Type locality: Van Bu [Son La Province, Vietnam].
Alycaeus vanbuensis – Inkhavilay et al. 2019: 13, fig. 4c–e.
Pincerna vanbuensis – Do and Nguyen 2022: 369, 372, figs 3a–e, 6e.
Pincerna (?) vanbuesis – Páll-Gergely 2023b: 269–272, figs 3h, i, 12, 14g.

##### Material examined.

**Figure 9.**
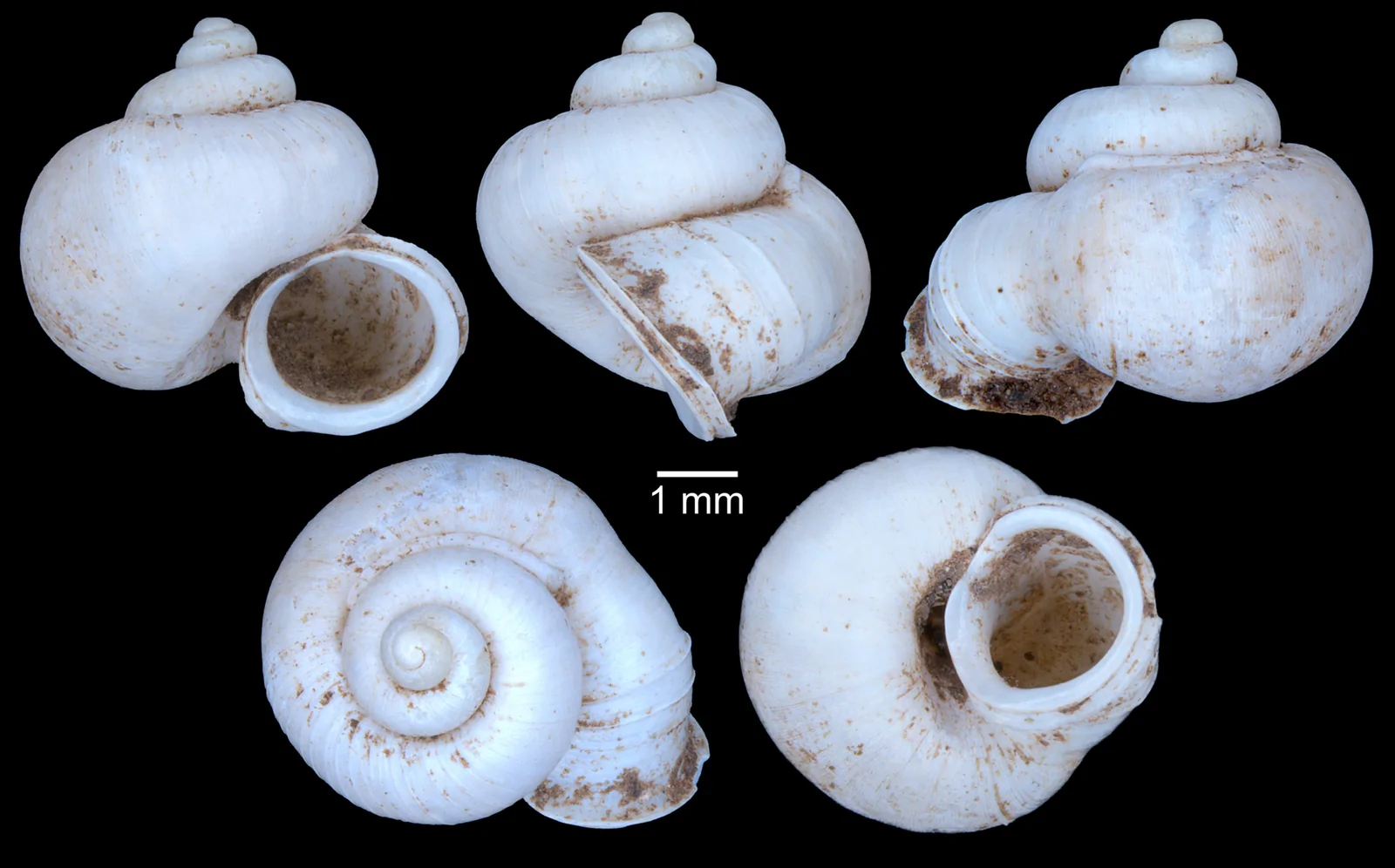
*Pincerna vanbuensis*, specimen CUMZ 14485 in different positions of the whole shell.

Laos • 5 shells; Houaphanh Province, Viengxay District, Ban Hang Long village; 20°27'38.7"N, 104°10'28.6"E; 840 m a.s.l.; 6 Oct. 2022; leg. K. Inkhavilay; CUMZ 14485 (Fig. 9).

##### Diagnosis.

R1 ~ 3¾ whorls with regular, fine radial ribs. R2 ~ ¼ whorl and nearly as long as R3; R2 with very fine and close-set ribs. R3 with less distinct ribs; apertural tube coiling slightly oblique to horizontal axis of the shell, expanding near the end of the aperture. Inner peristome blunt, slightly protruding. Outer peristome thickened, multilayered, expanded, slightly reflected. Umbilicus mostly closed by the expanded columellar extension of outer peristome.

##### Differential diagnosis.

See Páll-Gergely (2023b: 270).

##### Distribution.

Southern China, northern Vietnam; Luang Prabang, Luang Namtha, Khammouane (Páll-Gergely 2023b), and Houaphanh provinces, Laos.

##### Remarks.

Samples from different localities are variable in shell size, density, and strength of R1 ribs, and the extent of close umbilicus in shells with narrower body whorl (Páll-Gergely 2023b). See earlier taxonomic treatment of *P. vanbuensis* in Do and Nguyen (2022) and Páll-Gergely (2023b).

#### Pincerna panhai

Taxon classificationAnimaliaArchitaenioglossaAlycaeidae

Jirapatrasilp & Inkhavilay sp. nov.

C104AF4B-16EB-53F6-9294-EA48BECAD50B

https://zoobank.org/F666AD34-A601-4FB7-A5F9-7C3B806537F5

Figs 10, 11

##### Type material.

**Figure 10.**
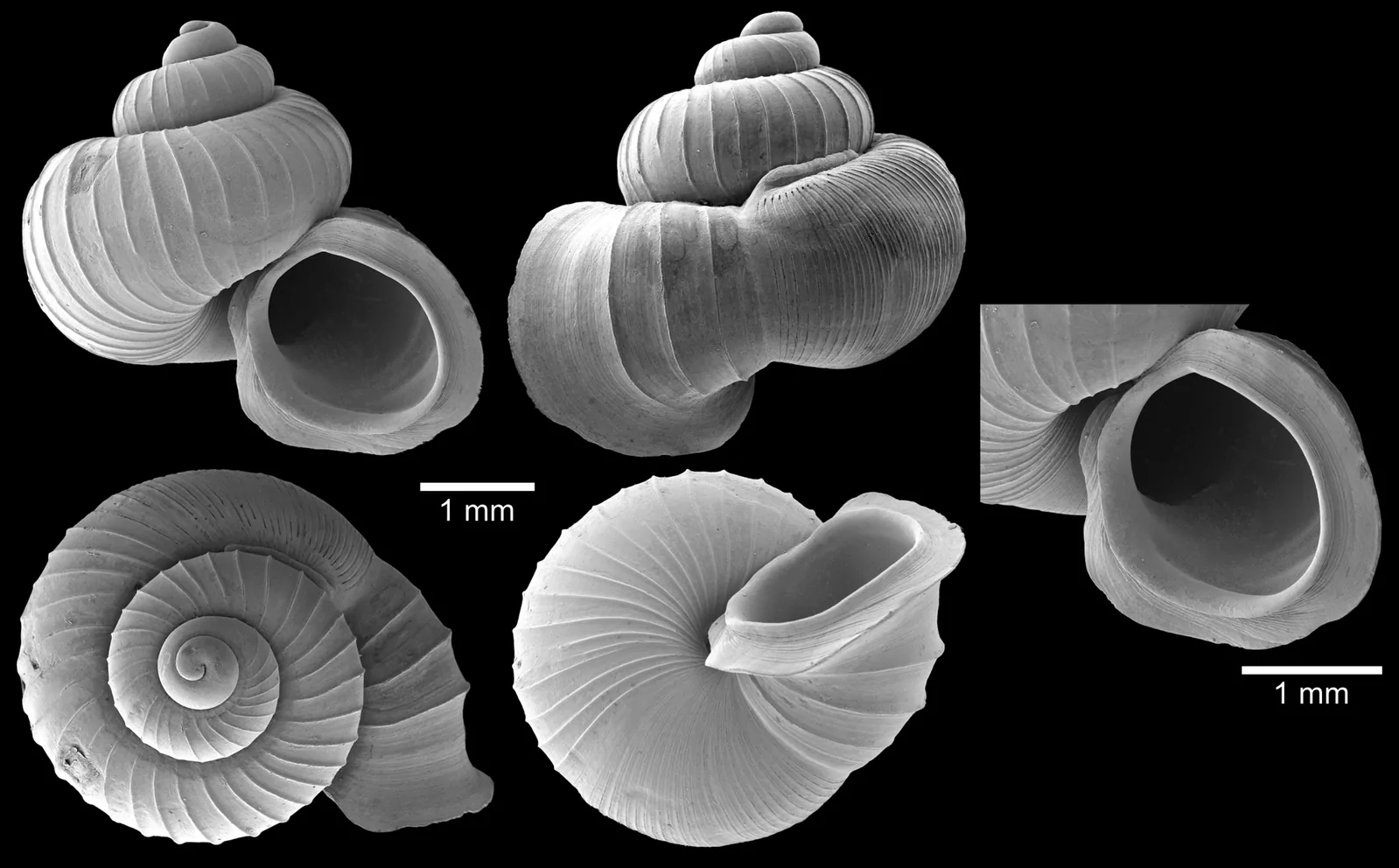
SEM images of *Pincerna panhai* sp. nov., holotype CUMZ 14486 in different positions of the whole shell and a close-up image of the aperture.

**Figure 11.**
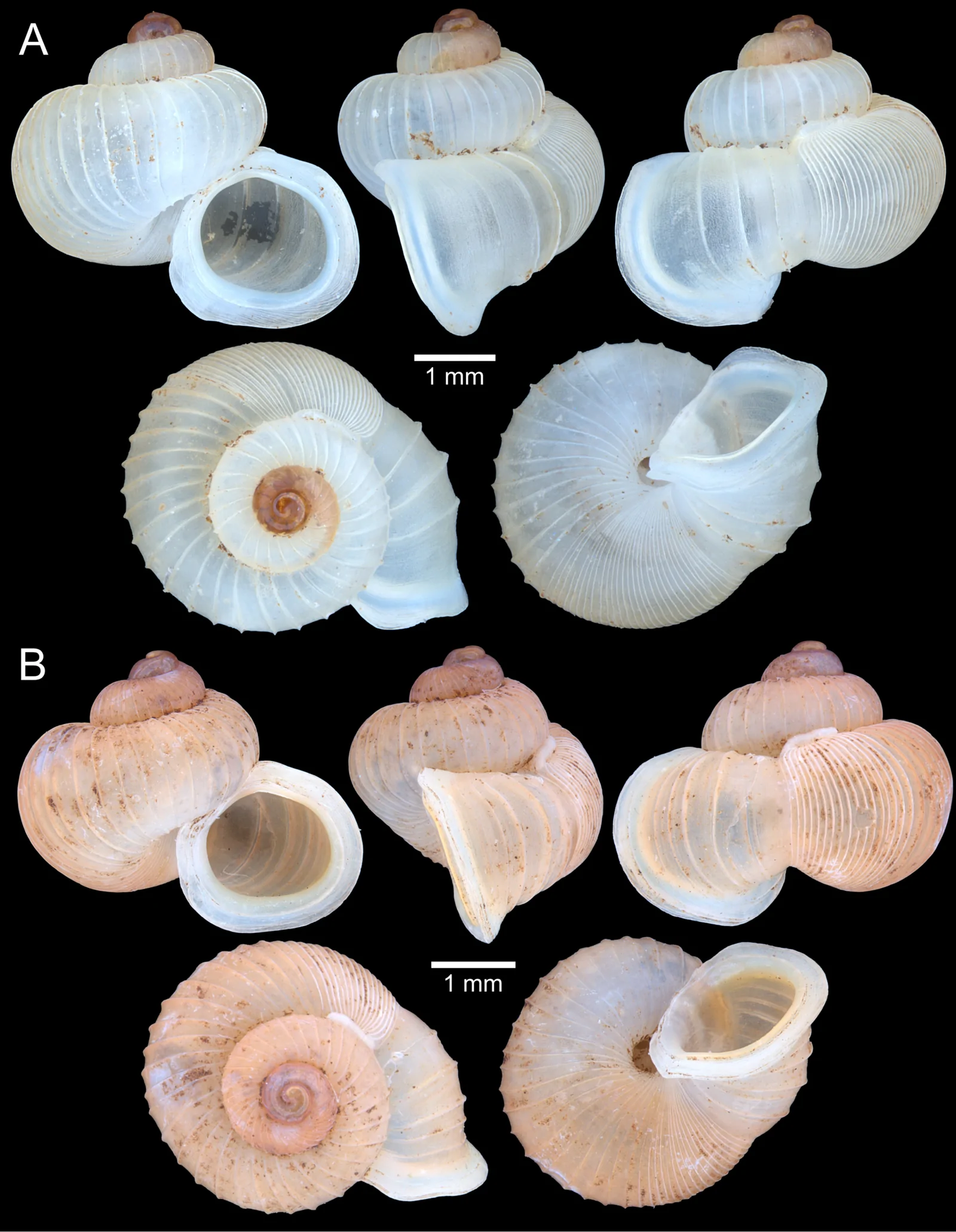
*Pincerna panhai* sp. nov. in different positions of the whole shell. **A**. Paratype CUMZ 14487; **B**. Specimen CUMZ 14488.

***Holotype***. Laos • 1 shell, SH = 3.9 mm, SW = 4.2 mm; Vientiane Province, Feuang District, unnamed limestone hill (loc. MF1); 18°40'29.2"N, 102°02'18.4"E; 320 m a.s.l.; 7 Oct. 2024; leg. CU-NUOL expedition team; CUMZ 14486 (Fig. 10). ***Paratypes***. Laos • 5 shells; same data as for holotype; CUMZ 14487 (Fig. 11A) • 2 shells; same data as for holotype; NHMUK 20260370 • 2 shells; same data as for holotype; SMF.

##### Other material.

Laos • 5 shells; Vientiane Province, Feuang District, limestone hill opposite to Xinxayyaram Temple (loc. MF8); 18°32'41.8"N, 101°58'47.0"E; 280 m a.s.l.; 8 Oct. 2024; leg. CU-NUOL expedition team; CUMZ 14488 (Fig. 11B) • 5 shells; Vientiane Province, Feuang District, Pha Reung (loc. MF14); 18°44'20.4"N, 102°07'02.9"E; 360 m a.s.l.; 10 Oct. 2024; leg. CU-NUOL expedition team; CUMZ 14489.

##### Diagnosis.

R1 ~ 1¾ whorls with regular, sharp radial ribs. R2 ~ ¼ whorl and nearly as long as R3; with ~ 25 thicker, blunter, and more close-set ribs. R3 ~ 3 ribs; apertural tube coiling more or less parallel to horizontal axis of the shell, expanding near the end of the aperture. Inner peristome blunt, slightly protruding. Outer peristome thickened, multilayered, expanded, not reflected. Umbilicus closed by the expanded columellar extension of outer peristome.

##### Differential diagnosis.

Among the ribbed species of *Pincerna*, *P. panhai* sp. nov. is similar to *P. costulosus* (Bavay & Dautzenberg, 1912) (Páll-Gergely 2023b: figs 8, 9) in its sharp, spaced radial ribs, and an umbilicus closed by the expanded columellar extension of outer peristome. However, the new species has a wider shell shape, a longer R2 (~ 25 vs 6–14 ribs), and R3 with sharper, more distinct ribs. The sharp, distinct R3 ribs of the new species match more with *P. acroptychia* Páll-Gergely, 2023 (Páll-Gergely 2023b: fig. 5a–e), but the new species has a wider shell shape, less close-set radial ribs, and a longer R2 (~ 25 vs 16 ribs). *Pincerna panhai* sp. nov. is also similar to *P. viginticostatus* Páll-Gergely, 2023 (Páll-Gergely 2023b: fig. 14a–f) in shell shape and R2 length, and a close umbilicus. However, the new species has sharper and less close-set radial ribs, and less R3 ribs (~ 3 vs 8 ribs).

##### Description.

Shell conical, globose, solid, translucent, whitish to pale crimson, SH 3.8–4.0 mm, SW 4.1–4.3 mm (*n* = 10). Shell outline round in apical view, spire ~ ¼ of shell height. Whorls ~ 4¼, last whorl large, round; spiral striation lacking on the entire shell. Protoconch high, ~ 1¾ whorls, glossy, smooth. Radial ribs strong, regular, starting from R1, continuing to the end of aperture. R1 ~ 1¾ whorls; with ~ 9 sharp ribs in ¼ whorl adjoining R2. Boundary between R1 and R2 distinct as R2 contains more close-set and thicker, blunter ribs than R1. R2 ~ ¼ whorl and nearly as long as R3; with ~ 25 ribs; ribs consistently thickened throughout. Boundary between R2 and R3 distinct as R3 abruptly expanded, and contains much less close-set and sharper ribs than R1 and R2. R3 with fine growth lines and ~ 3 ribs; coiling more or less parallel to horizontal axis of the shell, expanding near the end of the aperture. Aperture round; peristome double with prominent outer peristome. Inner peristome blunt, slightly protruding. Outer peristome thickened, multilayered (visible in lateral view), expanded, not reflected. Umbilicus closed by the expanded columellar extension of outer peristome. Operculum thin, translucent; multispiral ridges not elevated.

##### Etymology.

The species name is in honour of Prof. Somsak Panha, a prominent Thai malacologist and our beloved professor, who supervised the taxonomic and systematic study of land snails and other terrestrial invertebrates in Laos, as well as other countries in mainland Southeast Asia.

##### Distribution.

Known only from the limestone areas in Feuang District, Vientiane Province, Laos.

##### Remarks.

The occurrence of regular, sharp radial ribs, the number range of R2 and R3 ribs, and a close umbilicus are consistent across all specimens of this new species, suggesting that these are important diagnostic characters.

## Discussion

This study provides examples of different alycaein genera and different congeneric species occurring in the same area. In one particular site in Feuang District, Vientiane Province (MF1 limestone site), three *Dicharax* species (*D. cristatus*, *D. pallgergelyi* sp. nov., *D. feuangensis* sp. nov.) co-occur with *Pincerna panhai* sp. nov. Another site in Feuang District (MF14, Pha Reung) yielded three species from three different genera (*Dicharax cristatus*, *Dioryx feuangensis* sp. nov., and *Pincerna panhai* sp. nov.). Other examples of collecting sites with different alycaein species/genera co-occurring together are Doi Chiang Dao, northern Thailand, with four *Dicharax* species (Jirapatrasilp et al. 2021), several sites in Bhutan with up to four species in two genera (Gittenberger et al. 2022, 2024), and Gunung Jebak Puyuh, Pahang, Peninsular Malaysia, with five species in three genera (Foon and Liew 2017). Co-occurrence of different congeneric land snail species and different confamilial genera in the same area is common and could be attributed to either by habitat suitability with unlimited resources, or interspecific competition. On the one hand, there are examples of diverse land snail communities which often exhibit low levels of niche partitioning, meaning multiple congeneric species and confamilial genera frequently share the same microhabitats, e.g., leaf litter, woody debris, or limestone outcrops, without excluding one another (Němec et al. 2021, 2023). On the other hand, diverse land snail communities could be an instance of morphological overdispersion, which is typically driven by interspecific competition, as suggested by the study on *Cyclophorus* land snail communities from limestone karsts in Vietnam (von Oheimb et al. 2018). Nevertheless, the co-occurrence of different congeneric land snail species could be assumed as a transient state, resulting from very recent or recurrent colonisation events, and later the competitive exclusion of one species over another might follow (Fehér et al. 2018).

The discovery of new land snail species from limestone-rich areas of Laos also illustrates how important the limestone karst ecosystem contributes to Lao biodiversity. Limestone karsts are mainly distributed in the northern and central regions, with an approximate area of 30,000 km^2^ accounting for 12.6% of the country (Waltham and Middleton 2000; Clements et al. 2006). However, researchers and explorers largely focus on the biodiversity of limestone karst areas in Khammouane Province in central Laos, and to a lesser extent around Vang Vieng in Vientiane Province. These sites yielded many species new to science, spanning from vascular plants, insects, land snails to reptiles and mammals (Kumar et al. 2016; Vanhdibay et al. 2023; Vongphayloth et al. 2024; Souvannakhoummane et al. 2026). Since 2013, the Lao-Thai researcher team has covered more limestone-rich areas throughout the country, notably in Houaphanh, Luang Phrabang, Luang Namtha and Xiangkhouang provinces, resulting in a higher number of new land snail species from Laos (Inkhavilay et al. 2016a, 2016b, 2024; Inkhavilay and Sutcharit 2024). Despite the significance of an extensive limestone belt and its biodiversity, most limestone karstic sites, especially those in Feuang District, Vientiane and Viengxay District, Houaphanh explored in this study, were not included within any of the Key Biodiversity Areas (KBAs) of Laos (Tordoff et al. 2012; Key Biodiversity Areas Partnership 2026). Significant conservation efforts are crucially needed for these overlooked pristine karstic ecosystems, recognised as one of the unique centers of biodiversity and endemism in mainland Southeast Asia.

## Supplementary Material

XML Treatment for Metalycaeus

XML Treatment for Metalycaeus heudei

XML Treatment for Dioryx

XML Treatment for Dioryx dongiensis

XML Treatment for Dioryx feuangensis

XML Treatment for Dicharax

XML Treatment for Dicharax cristatus

XML Treatment for Dicharax pallgergelyi

XML Treatment for Dicharax feuangensis

XML Treatment for Pincerna

XML Treatment for Pincerna vanbuensis

XML Treatment for Pincerna panhai

## Additional information

### Artificial Intelligence (AI) use

The authors accept full responsibility for the content of the manuscript, including the disclosure of any use of AI.

No AI tools were used in the preparation of this manuscript.

### Author ORCIDs

P. Jirapatrasilp https://orcid.org/0000-0002-5591-6724

K. Inkhavilay https://orcid.org/0009-0007-1060-0885

T. Janjai https://orcid.org/0009-0007-1524-9668

E. Jeratthitikul https://orcid.org/0000-0002-3477-9548

N. Likhitrakarn https://orcid.org/0000-0002-1306-317X

R. Srisonchai https://orcid.org/0000-0002-7142-0999

C. Sutcharit https://orcid.org/0000-0001-7670-9540

### Data availability

All of the data that support the findings of this study are available in the main text.
